# Phospholipids and Sphingolipids in Osteoarthritis

**DOI:** 10.3390/biom15020250

**Published:** 2025-02-08

**Authors:** Juergen Steinmeyer

**Affiliations:** Laboratory for Experimental Orthopaedics, Department of Orthopaedics and Orthopaedic Surgery, Justus Liebig University, 35392 Giessen, Germany; just-oa@online.de

**Keywords:** phospholipids, sphingolipids, osteoarthritis, inflammation, synovial fluid, serum, plasma

## Abstract

Many studies now emphasize the intricate relationship between lipid metabolism and osteoarthritis (OA), a leading cause of disability. This narrative review examines alterations in the levels of phospholipids (PLs) and sphingolipids (SLs) in synovial fluid (SF), plasma, serum, and articular tissues; discusses their role in joint lubrication, inflammation, and cartilage degradation; and describes their potential as diagnostic markers and therapeutic targets. Key findings include stage-dependent elevated levels of specific PLs and SLs in the SF, blood, and tissue of OA patients, implicating them as possible biomarkers of disease severity and progression. Studies suggest that beyond the involvement of these lipids in joint lubrication, individual species, such as lysophosphatidylcholine (LPC) 16:0, lysophosphatidic acid (LPA), ceramide-1-phosphate (C1P), and sphingosine-1-phosphate (S1P), contribute to pain, inflammation, and degradation of joints through various signaling pathways. Cross-species comparisons suggest that dogs and mice experience similar lipidomic changes during OA as humans, rendering them valuable models for studying lipid-related mechanisms. PLs and SLs in SF appear to originate primarily from the synovial blood capillaries through diffusion. In addition, lipids that are produced locally by fibroblast-like synoviocytes (FLSs) are influenced by cytokines and growth factors that regulate the biosynthesis of PLs for joint lubrication. Emerging research has identified genes such as UGCG and ESYT1 as regulators of lipid metabolism in OA. Further, we examine the suitability of lipids as biomarkers of OA and the potential of targeting the PL and SL pathways to treat OA, emphasizing the need for further research to translate these findings into clinical applications.

## 1. Introduction

Osteoarthritis (OA), which is the predominant type of arthritis, significantly contributes to chronic pain and disability worldwide, the prevalence of which is rising sharply due to aging populations and increasing obesity rates. A recent study by the Global Burden of Disease Study in 2021 highlights the significant global impact of OA, projecting nearly 1 billion cases by 2050 and emphasizing the urgent need for preventive strategies and equitable access to effective treatments [1]. The prevalence of OA has climbed 132% worldwide from 1990–2020, and in 2020, approximately 595 million people were affected, representing 7.6% of the world’s population [1].

Recent research highlights the intricate connection between lipid metabolism and OA. Cholesterol, fatty acids, and apolipoproteins (Apos) are pivotal in the onset and progression of this degenerative joint disease; however, changes in lipid metabolism may serve as both a risk factor for and a result of OA [2,3,4,5,6,7]. Elevated cholesterol levels correlate with a higher risk and severity of OA, wherein OA patients typically exhibit increased serum LDL cholesterol compared with healthy individuals [2,8]. The influence of fatty acids on OA is complex: omega-3 fatty acids are noted for their protective effects, whereas the function of omega-6 fatty acids remains debated [9,10,11,12]. Further, genetic factors that affect cholesterol metabolism, such as variations in the CH25H-CYP7B1-RORα axis and the LRP3 gene, have been linked to the pathogenesis of OA [6,7,13]. Overall, the interplay between cholesterol, fatty acids, and Apos highlights the metabolic dimensionality of OA. Understanding these relationships can aid in the development of targeted therapies to manage or prevent the progression of OA by addressing metabolic dysfunctions [2,9,12,14].

While continuing to study the role of cholesterol and fatty acids in OA, emerging research suggests that focusing on phospholipids (PLs) and sphingolipids (SLs) will provide new insights into its pathogenesis and potential treatments. As discussed in this review, these lipids are integral to the composition of synovial fluid (SF); cellular membranes; cellular signaling; and the metabolism of articular cells, such as chondrocytes and fibroblast-like synoviocytes (FLSs). Directing efforts toward PLs and SLs may identify promising avenues for understanding the mechanisms of OA and developing novel therapeutic and diagnostic approaches. Thus, their roles in these processes may be key to unlocking new strategies for managing OA.

## 2. Methods

Search terms used in PubMed database and Google Scholar to identify articles published up to December 2024 included various combinations of the following keywords: phospholipid, sphingolipid, osteoarthritis, rheumatoid arthritis, serum, plasma, synovial fluid, inflammation, pain, lubrication, preclinical, cell culture, cartilage, chondrocytes, fibroblast-like synoviocytes, FLS. Additionally, the author’s own knowledge and work in the field were included when relevant. Focus was placed on the specific role that phospholipids and sphingolipids can play in the diagnosis and treatment of OA. The inclusion criterion for articles analyzed in this study was articles published in English only. References cited in the identified articles were individually examined for relevance and selected where appropriate. Exclusion criteria included articles published in a language other than English.

## 3. Chemical Structure and Biosynthesis of Phospho- and Sphingolipids

PLs and SLs are two essential classes of lipids that are critical in the structure and function of the cell membrane; however, they differ significantly with regard to their chemical structures and biosynthetic pathways. Whereas PLs are built on a glycerol backbone and synthesized primarily in the endoplasmic reticulum (ER), SLs are generated using a sphingoid base and require the ER and Golgi apparatus for complete synthesis.

PLs consist of a glycerol backbone, two fatty acid chains, and a phosphate group that has been modified by an alcohol group, giving them an amphipathic nature with hydrophilic heads and hydrophobic tails. Their synthesis begins with the formation of phosphatidic acid, a precursor that is created by adding fatty acyl-CoA to glycerol 3-phosphate, catalyzed by glycerol 3-phosphate acyltransferase and acylglycerol-3-acyltransferase [15]. This phosphatidic acid can be converted to diacylglycerol, which serves as a substrate for synthesizing phosphatidylcholine (PC) and phosphatidylethanolamine (PE) via the CDP-choline or CDP-ethanolamine pathway—separate branches of the Kennedy pathway—or the phosphatidylserine decarboxylation pathway [16]. This process occurs primarily in the ER, with some steps taking place in mitochondria, particularly for PE synthesis [15]. PC and PE are the most abundant PL classes in eukaryotic cells. Lysophosphatidylcholine (LPC) is derived from PC through phospholipase A2, and phosphatidylethanolamine-based plasmalogens (PE Ps) are defined by the presence of a vinyl-ether bond at the sn-1 position and an ester bond at the sn-2 position. Their head groups comprise serine, glycerol, and inositol, characterizing phosphatidylserine (PS), phosphatidylglycerol (PG), and phosphatidylinositol (PI), respectively.

SLs are hallmarked by their sphingoid base backbone, typically an 18-carbon amino alcohol, called sphingosine. The SL family includes sphingomyelin (SM), ceramide (Cer), sphingosine, sphingosine-1-phosphate (S1P), and Cer-1-phosphate (C1P). The synthesis of ceramide through the de novo pathway starts with the reaction between serine and palmitoyl-CoA, producing 3-keto-sphinganine. This process is catalyzed by the enzyme serine palmitoyltransferase [17]. The resulting intermediate is reduced to sphinganine, which is then acylated by ceramide synthase to form dihydroCer and subsequently desaturated to Cer. Cer serves as a precursor for more complex SLs, including SMs and glycosphingolipids. The initial steps of the synthesis of complex SLs occur in the ER, but this process is completed in the Golgi apparatus [17]. SM is the predominant SL and is synthesized from two precursors: Cer and PC. Further, Cer can be phosphorylated to C1P or metabolized by ceramidases to form sphingosine, which can be further phosphorylated by sphingosine kinases to generate S1P [18].

The synthetic pathways of PLs and SLs diverge significantly with regard to their initial substrates and enzymatic processes. Whereas PLs utilize glycerol 3-phosphate as their basic building block, the synthesis of SLs is initiated by the condensation of serine with palmitoyl-CoA [17]. Their enzymatic cascades also differ, wherein PLs involve acyltransferases and transferases that are related to glycerophosphate derivatives versus SLs, which rely on such enzymes as serine palmitoyltransferase and Cer synthases. Notably, the intracellular sites of synthesis vary—PLs are generated primarily in the ER, whereas SLs initiate in the ER but convert into their complex forms in the Golgi apparatus [17,19,20].

## 4. Phospholipid-Related and Sphingolipid-Related Diseases

Alterations in the levels of PLs and SLs in various body fluids have been associated with several metabolic and inflammatory diseases. These changes have been detected in such tissues as the liver, brain, and joints and in body fluids, including plasma, serum, SF, and cerebrospinal fluid, providing insights into disease mechanisms and potential diagnostic markers. Recent research has identified links between lipid alterations and a variety of diseases, ranging from metabolic disorders to neurodegenerative and inflammatory diseases. Table 1 gives an overview of the levels of PL and SL in various body fluids and tissues that are disrupted in common diseases, including atherosclerosis, Parkinson’s disease, Alzheimer’s disease, multiple sclerosis, non-alcoholic fatty liver disease, type 2 diabetes, multiple sclerosis, and joint diseases such as OA and rheumatoid arthritis (RA).

However, given the heterogeneity of diseases in which PL and SL levels are altered, the research results that have been obtained to date on such patterns may have disparate (patho)physiological, diagnostic, and therapeutic implications. Thus, our review focuses on OA and the accompanying disruptions in PL and SL metabolism, providing valuable insights into PL and SL synthesis, their relationship with the pathophysiology of OA, and their potential diagnostic use as biomarkers or therapeutic targets.

## 5. Altered Levels of Phospholipids and Sphingolipids in Human Osteoarthritis

Hills et al. [72] had recognized the importance of PLs in joint lubrication by 1998, emphasizing the critical function of surface-active PLs as boundary lubricants in natural and prosthetic joints [73,74,75,76]. They hypothesized that deficiencies in surface-active PLs increase friction and wear in load-bearing joints, linking such shortages to joint diseases such as OA [73,75]. The multifunctional nature of surface-active PLs allows their purview to extend to sites beyond joints to other tissues in the body with sliding surfaces, constituting potential clinical applications for treating various conditions and improving the longevity of prosthetic implants [73,74,75]. For example, Hills et al. [72] demonstrated that intra-articular steroid injections into the right radiocarpal joint increase the content of PLs in the SF of horses, based on chloroform extraction and quantification of phosphorus content of PLs in their entirety. The authors of [72] hypothesized that the corticosteroid-induced elevations of PLs in SF explain in part the therapeutic benefit of this drug class in joint diseases, although the extant higher PL and SL levels in the SF of patients with OA or RA were unknown at the time. Notably, Hills et al. [75] proposed that type B synoviocytes, also called fibroblast-like synoviocytes (FLSs), produce surface-active PLs that lubricate natural and prosthetic joints.

Since the seminal studies by Hills et al. [72,73,74,75,76], several groups have reported extensive and groundbreaking results on lipidomics in OA. These findings are reviewed briefly below to illustrate the current state of knowledge and their potential pathophysiological, diagnostic, and therapeutic implications. Table 2 summarizes the major findings from human studies in chronological order.

### 5.1. Human OA Synovial Fluid

In 2013, Kosinska et al. reported a comprehensive lipidomic analysis of a broad range of PL classes in the SF of patients from the so-called Giessen OA cohort, which consisted of nine healthy postmortem controls, 17 patients with early-stage OA, and 13 patients with late-stage OA knees [45]. Using electrospray ionization tandem mass spectrometry (ESI-MS/MS), the study identified 130 lipid species distributed across eight classes: PC, LPC, PE, PE P, PS, PG, SM, and Cer. Compared with controls, the SF from eOA and lOA patients contained elevated levels of many PL species. For instance, the median PC concentrations were 2.7-fold higher in early OA and 5.4-fold higher in late OA versus controls. Stage-dependent differences were reported for nearly 50% of all PL species, which differed between early and late OA. Our study suggested that the altered PL composition in OA affects joint lubrication and contributes to cartilage damage, highlighting the role of lipids in its pathogenesis.

In 2014, our group reported on SLs in SF from the same cohort, particularly SM and Cer species, as well as minor glycerophospholipids [46]. Based on liquid chromatography–tandem mass spectrometry (LC-MS/MS) and ESI-MS/MS analyses, SM levels in SF were elevated 2.4-fold in early OA and 4.4-fold in late OA, and total Cer levels increased 2.0-fold and 3.9-fold, respectively, compared with normal SF [46]. The authors ultimately identified 41 and 48 SL species that were significantly higher in the SF of patients with early OA and late OA, respectively, compared with normal SF. This study suggested that SLs influence inflammation and repair processes in joints, representing potential biomarkers of the severity of OA.

Through targeted metabolomic profiling of SF, a 2014 study by Zhang et al. revealed distinct metabolic subgroups in OA patients, highlighting the metabolic heterogeneity of OA and the potential role of lipid metabolism in its progression [47]. Two overarching, metabolically distinct phenotypes were identified among 80 OA patients [47]. One group was characterized by significantly higher concentrations of 37 acylcarnitines but lower free carnitine levels compared with the second group. The latter was subdivided into Groups B1 and B2. Subgroup B2 was characterized by higher levels of 75 glycerophosphollipids, consisting of 69 PCs and six LPCs, the fold-increases of which ranged from 1.58 to 1.83; nine SLs were also elevated in this subgroup by approximately 1.7-fold relative to B1, comprising six SMs, three hydroxysphingomyelins, one biogenic amine, and one acylcarnitine [47]. These subgroups were not associated with any known confounders, such as age, sex, BMI, and comorbidities [47]. Thus, glycerophospholipids significantly differentiated OA subgroups, particularly within Group B.

A 2021 lipidomic study by Rocha et al. also demonstrated higher SF levels of several PCs, PSs, and PIs in seven early-stage and eight late-stage knee OA patients compared with four non-OA control donors [48]. By targeted multiple reaction monitoring-mass spectrometry (MRM/MS) for high-sensitivity quantification of specific PLs, this group reported stage-specific differences, wherein the SF of early-stage OA patients had higher PC levels than those with late-stage OA, which correlated negatively with cartilage damage [48]. Notably, the authors stratified early-stage OA patients into two distinct subgroups based on their PL profiles in SF [48].

In a lipidomic study by Bocsa et al. (2022), the metabolic profile of SF from human OA knees was analyzed, identifying stage-dependent differences between early-stage and late-stage OA and highlighting potential biomarkers and metabolic pathways that are involved in disease progression [49]. Through untargeted metabolomic profiling by LC-MS, SF samples from the knee joints of 12 early-stage OA and 19 late-stage OA patients, based on the radiographic Kellgren–Lawrence (K-L) scale, were analyzed. The study identified 43 metabolites in these SF samples, nine of which were consistently present in at least 25% of patients across the early and late stages: four PLs—PC (20:0/18:2), PC (18:0/20:2), SM, and Cer (d18:1/20:0)—three purine metabolites, the gonadal steroid hormone estrone 3-sulfate, and heme. Most metabolites were enriched in the late-stage OA group, except for Cer (d18:1/20:0), which was higher in early-stage patients [49].

Khoury et al. (2023) identified specific choline-containing lipids, particularly LPC species, as potential biomarkers for chronic joint pain across several joint diseases, including OA [50]. This research used a comprehensive approach to analyze lipids in SF from 10 postmortem controls and 50 patients with various joint diseases, including 18 OA patients. Lipids were analyzed by high-resolution MS with ESI, and tandem MS was performed to confirm lipid structures. In total, 17 PCs, nine LPCs, and six plasmalogen PCs (PC Ps) were quantified. LPC 16:0, LPC 18:0, and the PC and PC P classes were observed at significantly higher concentrations in the SF of OA patients versus controls. These findings align with previous research by the same group [77] on the role of LPC 16:0 in chronic joint pain but constitute a broader lipidomic analysis across multiple joint diseases. Other studies have also analyzed LPCs in joint diseases but have looked at all lipid classes. For example, Kosinska et al. (2013) noted significantly elevated levels of 57 PL species, including 13 LPC species, in the SF of early OA patients versus postmortem controls but did not highlight LPC 16:0 or LPC 18:0 specifically [45]. Khoury et al. [50] did not find any significant correlation between lipid levels and patient gender or body mass index (BMI). A negative correlation was observed between age and the levels of certain lipids, such as LPC 16:0, LPC 18:0, and PC species, suggesting that lipid concentrations decrease with age.

### 5.2. Human OA Plasma or Serum

In 2010, Castro-Perez et al. published a comprehensive lipidomic analysis of 284 lipids using ultra-performance liquid chromatography–time-of-flight mass spectrometry (UPLC-TOF-MS) that revealed significant changes in the PL profiles of plasma from 59 female patients with knee and hip OA in the Dutch CHeCK cohort [51]. Subjects were classified based on radiological features of OA in the knee and hip joints per the K-L grading system; 26 of the included patients had a K-L grade of O and served as controls [51]. The lipidomic analysis allowed significant separation between groups, i.e., control, early OA, and moderate OA. As a result, arachidonic acid emerged as an important biomarker [51]. The 284 fatty acids, lipids, and triglycerides that were identified included 65 PCs, 43 SMs, 40 PSs, 22 LPCs, 7 PIs, 4 PEs, 3 PGs, and 2 LPEs. The 10 most common lipids belonged to the PC, SM, and triacylglyceride lipid classes. The study’s findings suggested the potential for the use of lipid profiles as biomarkers for the progression and diagnosis of OA.

The ratio of LPCs to PCs has emerged as a promising biomarker for knee OA, with potential applications in predicting disease progression, treatment response, and structural changes in joints. Three key papers from 2016 and 2019 examined this ratio with regard to knee OA [52,53,54]. By targeted metabolomic profiling, applied to serum or plasma samples from clinical trial cohorts and longitudinal studies using a commercially available kit and MS, they consistently demonstrated the potential of the LPC:PC ratio as a tool for predicting OA outcomes and personalizing treatment approaches. As a result of the analysis, this biomarker was significantly linked to the following:

- Advanced OA that required TKR, wherein patients with an LPC:PC ratio ≥ 0.09 were 2.3 times more likely to undergo TKR over a 10-year period [52]. The discovery cohort included 64 TKR cases and 45 controls, whereas the replication cohorts comprised a cross-sectional group of 72 TKR cases and 76 controls; a longitudinal cohort contained 158 subjects [52].
- A symptomatic response to anti-inflammatory treatments, including licofelone and naproxen, wherein knee OA patients with an LPC:PC ratio ≥ 0.088 were 2.93 times more likely to respond positively [54]. This finding suggests that the serum LPC:PC ratio is a valuable marker for predicting the response to anti-inflammatory treatments in knee OA patients, potentially aiding in personalized treatment approaches. The post hoc study in which these findings were presented examined 158 knee OA patients with a K-L grade of 2–3 who were part of a 24-month clinical trial that compared the effects of licofelone and naproxen [54].
- A loss of cartilage volume over time, particularly in the lateral compartment, as measured by MRI [53]. The post hoc study above [54] analyzed 139 knee OA patients with a K-L grade of 2–3, focusing on cartilage volume loss over 24 months by MRI. A significant association was found between the serum ratio of LPC 18:2 to PC 44:3 and loss of cartilage volume in the lateral compartment [53].

These findings highlight the potential of the LPC:PC ratio as a versatile tool for stratifying risk, treatment personalization, and disease progression monitoring in patients with knee OA. The conversion of PC to LPC, mediated by phospholipase A2 (PLA2), seems to play a key role in the pathology of osteoarthritis. Zhai et al. (2019) found that PLA2G5, a PLA2 family member, is significantly overexpressed in OA cartilage and synovial membrane compared with controls. This overactivation of the PL metabolic pathway correlates with increased levels of inflammatory markers, such as IL-6, suggesting a mechanistic link between altered lipid metabolism and OA progression [53].

A metabolomic study by Tootsi et al. (2020) revealed significant alterations in the amino acid, biogenic amine, and lipid profiles of serum from patients with severe OA versus controls, providing insights into the complex interplay between chronic inflammation, oxidative stress, and disease progression [55]. Serum was obtained from 70 OA patients with end-stage knee and hip OA and a cohort of 82 age-matched and gender-matched individuals as symptom-free controls. A commercially available kit was used to perform the targeted metabolomic profiling, which quantified 186 metabolites, including amino acids; biogenic amines; and complex lipids, such as PCs, LPCs, and SMs. The serum levels of eight PC species were lower in OA patients, particularly those that contained polyunsaturated fatty acids, whereas two PC species and SM 20:2 were detected at higher levels in these patients [55]. Conversely, LPC 20:4 was elevated in OA patients. LPCs are bioactive lipids with proinflammatory properties and the potential to induce cell death. This study provides novel insights into the metabolic shifts that are associated with severe OA, emphasizing systemic inflammation and oxidative stress [55].

In 2022, a lipidomic study by Loef et al. examined the link between plasma lipid profiles and OA severity using a large lipidomics platform [56]. Plasma samples from 216 participants with knee OA were obtained from the Applied Public–Private Research enabling OsteoArthritis Clinical Headway cohort and were analyzed for triacylglycerols, diacylglycerols, cholesteryl esters, ceramides, free fatty acids, SM, PLs, and oxylipins. The higher-order lipids were quantified on the high-throughput Lipidyzer™ platform, and oxylipins were analyzed by LC-MS/MS. The association between lipidomics and OA was weak, in that the lipidomic profiles explained only a small portion of the variance in radiographic knee (3%) and hand OA severity (2%) but accounted for the greater variance in hand pain (12%) and function (6%). The authors concluded that lipids have a role in hand pain but have limited influence on radiographic OA severity. However, the study did not include a healthy control group, but analyzed the association between lipid profiles and OA severity within the patient cohort itself [56].

A recent study by Li et al. (2024) identified distinct lipid profiles in serum and urine samples—signatures that could serve as biomarkers for diagnosing seropositive and seronegative RA and parsing the subtle alterations in lipids that are associated with OA [57]. This study used serum and urine samples from 111 RA patients, 45 OA patients, and 25 healthy controls. Extracted lipids were analyzed by untargeted lipidomics using ultra-performance liquid chromatography coupled with ESI-MS/MS, enabling the authors to identify and quantify various lipid classes, including PC, LPC, SM, and Cer [57]. As a result, a panel of 10 serum lipids and three urinary lipids were identified as potential biomarkers of RA. The serum lipid panel demonstrated high diagnostic accuracy, with 79% accuracy, 71% sensitivity, and 86% specificity in distinguishing RA from OA and healthy controls [57]. Unlike in RA, in which many lipids underwent significant changes compared with controls, few lipids were differentially expressed between OA and healthy controls—SM t39:0 was significantly upregulated, and LPC-ether (LPC-O 18:0) was downmodulated in OA patients versus healthy controls [57]. These findings implicate SLs in cartilage degradation and synovial inflammation, whereas changes in LPCs may reflect a distinct inflammatory profile in OA. In urine samples, no specific lipids differed significantly between OA and controls, suggesting that urinary lipid profiles are less able to differentiate OA from healthy controls than serum profiles. The intermediate lipid levels that were observed in OA patients, lying between those of RA patients and healthy controls, reflect the less aggressive inflammatory nature of OA. This finding is supported by the 2013 and 2014 studies by Kosinska et al., which also found that the concentrations of PLs and SLs were highest in the SF of RA patients compared with OA and healthy controls [45,46].

### 5.3. Human OA Plasma/Serum and Synovial Fluid

The relationship between blood plasma and SF metabolite concentrations in OA patients was the subject of a 2015 study by Zhang et al. [58]. Paired plasma and SF samples were obtained from 69 late-stage OA patients and analyzed using a targeted metabolomic approach by ESI-MS and a commercially available kit, quantifying 168 metabolites, including 87 glycerophospholipids and 11 SLs. This study observed only modest correlations between plasma and SF metabolite concentrations in OA patients, yielding an average Spearman’s rank correlation coefficient (ρ) of 0.23 for absolute concentrations [58]. Both the glycerophospholipid and SL classes showed slightly higher correlations, with average ρ values of 0.26 and 0.25, respectively. Only eight of 168 metabolites had significant correlations of *p* ≥ 0.45, comprising four amino acids (glycine, leucine, valine, isoleucine), three glycerophospholipids (e.g., PC 36:0), and creatinine [58]. Notably, the metabolite ratios demonstrated stronger correlations (average ρ = 0.29), with 4018 ratios achieving ρ ≥ 0.52 [58]. Further, lipid-related metabolite ratios, particularly those that involve PLs and SLs, exhibited even stronger correlations (ρ = 0.6–0.85) [58]. These results suggest that although absolute plasma concentrations of metabolites do not fully reflect joint-specific processes, metabolite ratios are more reliable indicators of systemic metabolic processes in OA.

A study from 2016 by the same group reported that altered PC metabolism is associated with OA and type 2 diabetes mellitus, identifying specific metabolites as potential biomarkers of their concurrence [59]. Targeted metabolomics by UPLC-MS, coupled with a commercial assay, was performed to measure PC metabolism in 54 paired SF and plasma samples from late-stage knee OA patients and plasma from 30 controls, matched for age, sex, and BMI in the discovery stage. A total of 143 plasma samples (72 from knee OA patients and 71 from controls, matched for age, sex, and BMI) were analyzed in the validation stage. This commercial kit enabled them to analyze 186 metabolites, including 90 glycerophospholipids. A 2-stage case–control study design involving discovery and validation cohorts was used to confirm the findings. The resulting metabolic profiles in SF and plasma made it possible to distinguish OA patients with metabolic syndrome from those without it [59]. Among the components of metabolic syndrome, type 2 diabetes was determined to be the primary driver of these metabolic differences, rather than obesity, hypertension, or dyslipidemia. The two PC species, PC O-34:3 and PC O-36:3, were identified as potential biomarkers for the concurrence of OA and diabetes, wherein their concentrations in SF correlated significantly with their plasma levels. These metabolites were significantly lower in affected individuals, suggesting their diagnostic and therapeutic relevance [59]. Further, certain PC species were inversely related to insulin resistance and triglyceride levels, indicating their broader role in metabolic regulation and implicating a possible mechanistic link between OA and type 2 diabetes. Thus, the study suggests that PC metabolism is a shared pathway that connects OA and diabetes and constitutes a potential therapeutic target for managing OA in patients with metabolic comorbidities.

A 2024 study by Stanciugelu et al. (2024) examined 33 patients, mostly with knee OA, and compared them with 20 control subjects who had bone fractures or ligament ruptures from accidents [60]. This study identified potential lipid biomarkers in both plasma and SF that could aid in the early diagnosis of OA. Plasma and SF samples were analyzed by UHPLC-QTOF-ESI+MS, a powerful tool for lipidomics with certain limitations, such as ionization bias and inconsistent quantitative accuracy. In plasma samples, 25 metabolites displayed area-under-the-curve values above 0.9, signifying outstanding diagnostic potential. Among the key biomarkers identified were phosphatidic acid PA 16:0/16:0, PA 34:0, PE 34:2, glucosylceramide, and PC 32:1. In SF, 20 metabolites exhibited area-under-the-curve values exceeding 0.8, most of which belonged to lipid metabolism [60]. These data align with previous research that has highlighted the importance of SF in evaluating metabolomic profiles in OA, given that it reflects the conditions in joint tissue directly. The altered lipid profiles in this study provided insights into disruptions in such metabolic pathways as plasmalogen synthesis, mitochondrial beta-oxidation, and SL metabolism. These pathways are involved in cellular energy production and inflammatory responses, which are crucial in the early development of OA [60]. Although the types of lipids that were detected between plasma and SF overlapped, certain lipids were unique to each biofluid. For example, glucosylceramide was prominent in plasma but not as significant in SF, whereas PS and PI species resided predominantly in SF. The identification of PL species as potential biomarkers corroborates earlier work by Kosinska et al., who reported increased concentrations of PLs in the SF of OA patients [45,46]. The study [60], which focuses on both plasma and SF samples, builds on the growing trend of using several types of biofluids to generate comprehensive metabolomic profiles of OA.

### 5.4. Human Articular Cartilage Surface

The composition of PLs of articular cartilage surface is crucial in joint lubrication and health, some research on which has shed light on their complex molecular structure and function. In Sarma et al. (2001), three major PL classes were identified on bovine articular cartilage surfaces [78]. In this study, bovine femoral condyles were immersed briefly in chloroform–methanol solution to extract surface-bound lipids [78]. PL classes were separated using thin-layer chromatography on silica gel-loaded paper and then quantified by a phosphate assay. Fatty acid profiles were analyzed by gas chromatography with flame ionization detection, and specific molecular species were identified by ESI-MS. The results showed that the surface of this bovine articular cartilage comprised three PL classes—41% PCs, 27% PEs, and 32% SMs—forming molecular layers that have been hypothesized to reduce friction under severe loading conditions [78]. These PLs contained a higher percentage of unsaturated (57%) versus saturated fatty acids (43%), of which oleic acid (C18:1) was the most prevalent across all PLs, followed by linoleic (C18:2), palmitic (C16:0), and stearic (C18:0) acids [78].

The unique composition and structure of these specific lipid layers are believed to contribute to the robust tribological properties of cartilage, wherein the hydrophilic heads adhere to the surface, and the hydrophobic tails extend outward to form a low-friction hydrocarbon layer [78]. This study emphasized the importance of a mixture of PLs, rather than a single type, for enhanced stability and lubrication but was limited to three major PL classes, potentially overlooking other minor but functionally significant lipids [78]. Additional research into lipid–protein interactions on cartilage surfaces may yield details into how PLs interact with other components such as lubricin and thus improve our understanding of this complex lubrication system. This approach will support the development of therapies that are based on the improved lubrication of cartilage defects and prostheses.

A study by Chen et al. (2007) revealed that unsaturated PCs are the dominant surface-active PL species on cartilage surfaces, challenging previous assumptions about joint lubrication [79]. Surface-active PLs were prepared from 10 bovine cartilage specimens by the Folch procedure, involving chloroform–methanol solvent extraction. Individual PC species in these samples were identified and quantified primarily by high-performance liquid chromatography [79]. The study identified four main unsaturated PC species: PC 36:4 (dilinoleoyl-phosphatidylcholine), PC 34:2 (palmitoyl-linoleoyl-phosphatidylcholine), PC 34:1 (palmitoyl-oleoyl-phosphatidylcholine, POPC), and PC 36:2 (stearoyl-linoleoyl-phosphatidylcholine). In contrast, the saturated PC 32:0 (dipalmitoyl-phosphatidylcholine, DPPC), previously believed to be the chief surface-active PL in joints, constituted only 8% of the total PC content. This finding is consistent with reports on surface-active PL profiles in other non-lung sites, such as the peritoneal and pleural cavities, where unsaturated PC species also predominate [79]. Sarma et al. (2001) had already indicated indirectly that unsaturated fatty acids abound on cartilage surfaces but did not identify specific unsaturated PC species [78]. The prevalence of unsaturated PCs on cartilage surfaces hints at their importance in joint lubrication and function. Chen et al. defined a more detailed molecular profile of cartilage surface-active PC species, offering new insights into joint physiology and potential therapeutic approaches for OA [79].

### 5.5. Human OA Synovium and Genes

A histology-based study by Rocha et al. (2021) established a distinct lipidomic profile in OA synovial membranes using advanced imaging techniques [61], analyzing synovial membranes from 13 OA, six RA, and 12 psoriatic arthritis patients and 10 controls. Control patients had undergone arthroscopy due to a post-traumatic event and had no secondary arthritis, rheumatic disease, or inflammatory joint effusion [61]. Using matrix-assisted laser desorption ionization–mass spectrometry imaging (MALDI-MSI), they identified 62 lipid species, 52 of which differed significantly between OA and control synovial membranes—the levels of PC, PE, PS, and PI species, fatty acids, and LPA 18:0 and LPA 18:1 were significantly elevated in OA tissues, whereas LPCs were decreased compared with control samples [61].

In addition, specific lipid species within the OA synovium assumed a unique spatial distribution. PC (18:1/18:1) localized predominantly to the hypertrophic synovial lining layer, and PS (18:0/20:4) was enriched around blood vessels and inflammatory cell aggregates in the sublining layer. Further, the study’s comparison of OA with RA and psoriatic arthritis revealed disease-specific lipid alterations between conditions [61]. OA tissues exhibited higher levels of certain PCs and SMs versus RA, whereas psoriatic arthritis tissues had elevated levels of PE Ps. This differentiation supports earlier findings in SF and plasma that have shown that lipid profiles can distinguish between various types of arthritis based on their inflammatory status [45,46,48,57]. The observed increase in LPA levels in OA synovium aligns with prior research on the involvement of LPAs in inflammation and pain regulation in OA [80]. LPAs have been linked to neuropathic pain and joint degeneration, rendering them potential targets for therapeutic interventions [80,81]. The study by Rocha et al. [61] adds important and novel insights into lipid localization within synovial tissue structures and provides a more comprehensive overview of tissue-specific lipid alterations in OA.

Using an innovative combination of bioinformatics analysis and machine learning algorithms, Li et al. (2024) found two hub genes, UGCG and ESYT1, to be differentially expressed in OA patients versus healthy controls [62]. Their study used two gene expression profiles from the peripheral blood mononuclear cells of 106 late-stage OA patients and 33 healthy controls and from synovium samples obtained from seven late-stage OA patients and seven postmortem joint-healthy controls. In total, 363 differentially expressed genes related to OA were identified and intersected with 776 lipid metabolism-related genes in the Molecular Signatures Database [62]. This step yielded nine differentially expressed genes that were associated with lipid metabolism in OA. Application of three machine learning algorithms, followed by further validation using an independent dataset, confirmed the significant differential expression of the UGCG (downregulated) and ESYT1 (upregulated) gene hubs in OA patients compared with healthy controls [62]. The UGCG gene encodes UDP-glucose ceramide glucosyltransferase, which catalyzes the initial crucial step in the biosynthesis of most glycosphingolipids. Loss of UGCG expression leads to elevated ceramide levels in chondrocytes, subsequently affecting cartilage matrix degradation and chondrocyte apoptosis [82]. ESYT1 encodes extended synaptotagmin-1, a protein that maintains mitochondrial homeostasis and adaptation; overexpression of E-Syt1 may result in lipid accumulation in mitochondria, inducing mitochondrial dysfunction and contributing to the development of OA [62]. Ultimately, both genes were proposed as potential diagnostic biomarkers for OA [62]. Targeting these genes or their related pathways could represent new therapeutic strategies for slowing or halting the progression of OA.

The systemic changes in PL and SL profiles that were observed in serum and plasma, as well as the identification of nine differentially expressed genes—including the aforementioned hub genes that are associated with lipid metabolism in OA—support the emerging view that OA is a metabolic disease that also affects non-weight-bearing joints [83]. In this context, Wu et al. have demonstrated correlations between serum fatty acids and OA severity but moderate associations between blood and SF profiles [84]. Also, a lipidomic study has reported differences in SF fatty acids between OA and control subjects, including a tendency toward lower levels of omega-6 fatty acids in the former [85]. This disparity could also contribute to the inflammatory environment that hallmarks the progression of OA [9].

Moreover, a study that queried the National Health and Nutrition Examination Survey (NHANES) database and performed Mendelian randomization analyses found that LDL cholesterol may actually be a protective factor for OA, whereas the function of high-density lipoprotein (HDL) cholesterol remains unknown, requiring further investigation [86]. In 2011, Gkretsi et al. [83] linked cholesterol metabolism to the development of OA, identifying serum cholesterol as a risk factor for OA [83] and observing the dysregulation of cholesterol-related genes in OA chondrocytes in gene expression studies [87]. A pivotal study by Choi et al. (2022) highlighted the CH25H-CYP7B1-RORα axis as a crucial regulator of cholesterol metabolism in OA, determining its function in cholesterol uptake via lectin-like oxidized low-density lipoprotein receptor-1; oxysterol production; and the activation of matrix-degrading enzymes, such as matrix metalloproteinase (MMP) 3, MMP13, and ADAMTS5, in chondrocytes [13]. Thus, identification of the involvement of the CH25H-CYP7B1-RORα axis in OA fills a gap in our understanding of how metabolic dysregulation contributes to its pathogenesis. Collectively, as our knowledge of lipid metabolism in OA deepens, it is becoming increasingly clear that lipids are crucial in disease progression.

### 5.6. Cross-Species Lipid Comparison in Healthy and OA Samples

In searching for an optimal animal model to study lipid-related cardiovascular disease, Kaabia et al. [88] reported an LC-HRMS-based lipidomic analysis of 106 lipids in 2018, including cholesteryl esters, di- and triglycerides, 47 PL species, and 11 SL species in the plasma of healthy humans compared with nine animal species. Their study observed that hamsters and, to a lesser extent, mice develop plasma lipid profiles that closely resemble those of humans, rendering them promising animal models for researching lipid-related diseases (Table 3) [88]. Robust, significant Pearson correlations of the relative abundance of the 106 identified lipids were noted between humans and hamsters (r = 0.816) and mice (r = 0.810), whereas other animal species had lower correlation coefficients with humans (r = 0.47–0.69), for example, dogs (r = 0.47) and horses (r = 0.62). Horses and other herbivorous species, such as cows, had lipid profiles that differed markedly from those of humans, primarily due to their characteristically low triglyceride levels. Their lipid fingerprints clustered away from humans based on principal component analysis, indicating dissimilar metabolic profiles to humans [88].

However, in selecting suitable animal models for OA, it is insufficient to have a similar lipidomic profile or high correlation with plasma from healthy humans—rather, it is necessary that OA induce comparable changes in blood and SF in the selected animal species as in OA patients. In OA animal models, several studies have noted altered levels of PLs and SLs in SF and plasma compared with healthy controls (Table 3). In comparing PL and SL levels in OA across humans, dogs, horses, and mice, species-specific differences in lipid profiles appeared, which may reflect variations in joint anatomy, metabolic processes, and disease progression. Understanding these lipidomic profiles is crucial for selecting appropriate animal models for OA research and developing targeted therapies.

Murine models of OA have provided valuable insights into lipid alterations that are associated with disease progression (Table 3). During destabilization of the medial meniscus, significant changes in plasma lipid profiles were observed in a surgical model that is widely used to induce OA in mice [89]. Cholesterol esters (CEs), particularly CE(18:2), CE(20:4), and CE(22:6), correlated positively with pain behavior and cartilage damage. Also, PC 36:2, PC 38:7, and SM(d34:1) were elevated in OA mice compared with controls and paralleled cartilage damage [89]. Using pathway analysis, SL metabolism was identified as a key pathway that is involved in altered lipid homeostasis in the OA mouse model [89]. However, no study has applied the same analytical method to compare PL and SL levels directly in plasma or SF of mice and humans with OA, as has been done for SF in dogs and horses [63,90].

Notably, murine cartilage exhibits unique lipid patterns during the development of OA. LPC species are enriched in the growth plate, an active site of mineralization [91]. Further, the ablation of bone-specific phosphoethanolamine/phosphocholine phosphatase, an enzyme that participates in bone mineralization, significantly alters the growth plate lipidome in mice, upregulating PC, LPC, SM, PE, and lysophosphatidylethanolamine (LPE) [91]. These findings highlight the complex interplay between lipid metabolism and the pathogenesis of OA in murine models. Thus, that the plasma lipidome in healthy mice correlates highly with that of humans, coupled with the evidence that OA in mice significantly alters their lipidomic profiles, suggests that mice represent a valuable animal model for studying OA. Further research is needed to compare them directly with OA patients.

**Table 3:** Cross-species lipid comparison in healthy and OA samples, and their relevance as models for studying OA.

Aspect	Humans	Dogs	Horses	Mice
Plasma lipidomic profile similarity to healthy humans [88]	Baseline	Moderate similarity (r = 0.47)	Moderate similarity (r = 0.62)	High similarity (r = 0.81)
OA-induced lipid changes	Significant increase in PC, LPC, and SM species in serum and SF during OA (see Table 2)	Similar increases in PC, LPC, and SM species as seen in human OA progression [63]	Lack of data on comparative OA studies; lower baseline PL and SL levels, and less polyunsaturated PL species in SF compared to humans [90]	PC 36:2, PC 38:7, SM(d34:1) elevated in plasma during OA [89]
Apolipoprotein levels	Higher levels of Apo B-100 in SF compared to horses [90]. Apo-AI levels in OA serum similar to healthy controls [4]	Higher Apo-AI levels in OA SF compared to controls [92]	Lower Apo B-100 levels (~32% of human levels) [90]. Apo-AI is elevated 2.1-fold in OA SF versus healthy controls [93]	N/A
Dietary influence on lipid profile	Omnivore, mixed diet influences lipid profile, particularly LDL levels	Meat-based diet increases polyunsaturated PCs	Herbivorous diet leads to lower triglyceride levels and distinct lipid profiles	N/A
Usefulness as a model for Studying OA	Baseline	Reliable model for early-stage OA	Less suitable due to lack of data on OA-induced PL changes and lower baseline PL and SL levels	Promising model for studying lipid alterations in OA but more data are needed on OA-induced changes

N/A: not available.

In a canine groove model of OA, we found that PL and SL levels in knee SF rose significantly during disease progression. In our study, total lipid content increased 2.6-fold compared with healthy controls, wherein PC, LPC, and SM were the predominant lipid classes in canine SF [63]. The lipid profile in canine OA closely mimicked that in early-stage human OA, characterized by elevated levels of PC (O), LPC, and SM species [63]. However, dogs had the highest content of PC species with polyunsaturated fatty acids, particularly arachidonoyl-containing PCs, which are linked to their meat-based diet [68]. Nevertheless, the similarity between PL and SL levels in dogs and humans suggests that dogs are a reliable model for studying lipid changes in early OA, providing valuable insights into disease mechanisms and potential therapeutic targets (Table 3) [63].

In our comparative study using joint-healthy SF, PLs were compared between equine and human SF, in which six lipid classes with 89 PL species were quantified [90]: PC, LPC, SM, PE, plasmalogen, and Cer. We found that equine SF has roughly half the PL concentration of human SF, with markedly lower median levels of LPC, PE, SM, and Cer species [68,90]. Compared with humans, horses have reduced SF levels of PL species of all classes with polyunsaturated fatty acids such as PC 36:4 and PC 38:4 [68,90]. These differences may reflect adaptations in horses to constant joint loading when standing and may be attributed to dietary habits, lower Apo levels, and changes in enzyme activity [94]. There are no comparative studies on OA-induced lipidomic changes in blood and SF in horses versus OA patients. Existing data show that the plasma and SF lipid profiles of healthy horses differ considerably from those of humans, rendering horses a less suitable species with which to model lipid-related diseases (Table 3).

Apos are structural proteins that stabilize lipoprotein particles and facilitate the transport of PLs and SLs in the blood and SF. In mammals, Apos are synthesized primarily in the liver and intestines, and their levels are influenced by diet. Apo B-100 and Apo B-48, which are found in domestic animals, are structurally identical to those in humans. However, the truncated form of Apo B is predominantly expressed in the intestines and liver of dogs and horses. In serum, Apo stabilizes lipoproteins, such as HDL (rich in Apo-AI), LDL, very low-density lipoprotein (VLDL), and intermediate-density lipoprotein (rich in Apo B-100) and carries PLs and SLs, including PC and SM. However, differences exist between species: dogs and horses have VLDL particles that contain Apo B-48, whereas humans predominantly have Apo B-100-bearing VLDLs (Table 3) [94]. Also, in humans, LDL carries most of the cholesterol, whereas HDL predominates in dogs and horses due to higher HDL-associated lecithin cholesterol acyltransferase activity in dogs and lower cholesteryl ester transfer protein activity in the two latter species [88,94].

In SF, Apo helps transport PLs and SLs to maintain joint lubrication. Lower Apo levels in horses correlate with reduced PL and SL concentrations compared with humans [63,90,94]. For example, horse knee joints contain approximately 32% of the Apo B-100 that is found in human SF [90]. Apo-AI concentration is 2.1 times higher in equine OA-SF than in healthy equine joints [93]. In dogs, Apo-AI is the major component of HDL particles, and its concentration in SF is significantly higher in those with OA [92]. The median Apo-AI concentration in the SF of OA dogs is 191 µg/mL, but such levels are undetectable in most control dogs [92], whereas 521 µg/mL were reported for human OA SF [4].

In summary, cross-species comparison of lipid profiles in healthy and OA joints revealed a number of similarities and differences (Table 3) [45,63,88,89,90]:

- PC is consistently the most abundant lipid class across humans, dogs, horses, and mice, and PC species are elevated in SF and/or serum during OA in humans, dogs, and mice.
- Humans and dogs experience similar increases in total lipid levels and elevations in PCs, LPCs, and SMs during the progression of OA.
- Dogs have higher plasma levels of PLs and total cholesterol than humans and other species, such as cows, horses, rats, and hamsters.
- The SF of healthy equine joints has a unique lipid profile, with lower overall PL concentrations and reduced levels of polyunsaturated PL species compared with humans.
- Many SM and PC species are elevated in human and canine OA SF and serum, whereas a mouse model of OA has shown increased plasma SM(d34:1), PC 36:2, and PC 38:7 levels associated with cartilage damage.
- Healthy mice have significantly lower plasma levels of certain lipid classes, such as SM and Cer, compared with healthy humans.
- Although not as closely aligned with human plasma lipid profiles as hamsters, mice still exhibit notable similarities, rendering them a useful model for studying lipid-related diseases.
- Apos are critical for the transport of PLs and SLs in serum and SF in humans, dogs, horses, and mice. Due to differences in nutrition, metabolism, and physiological needs, there are species-specific differences in Apo proteins. Humans generally have higher levels of Apo and lipids that are bound to them, whereas horses have lower levels of Apo and thus PLs and SLs.

Although there are common trends with regard to the elevation of certain lipids, such as PCs and SMs, between species during OA, each animal model bears a distinct lipidomic profile that reflects its unique dietary habits, joint anatomy, and metabolic processes. These differences highlight the importance of considering species-specific lipid alterations when translating OA research findings across animal models and into clinical applications.

## 6. Origin of Phospholipids and Sphingolipids in Synovial Fluid

SF contains PLs and SLs, which originate in part from synovial blood vessels and local cellular synthesis. Also, changes in lymphatic vessels may influence the composition of these lipids in SF.

### 6.1. Blood Vessels

A wide variety of PLs and SLs, essential components of cell membranes, exist in the human body. Early studies quantified nearly 600 distinct lipid molecular species in human plasma, encompassing the six main mammalian lipid categories: fatty acyls, glycerolipids, glycerophospholipids, SLs, sterol lipids, and prenol lipids [95,96,97]. However, the exact number depends on the analytical techniques used and the resolution of the lipidomics methods applied. Advanced mass spectrometry methods continue to uncover additional lipid species, further expanding our understanding of the plasma lipidome [98]. Studies have identified over 160 distinct glycerophospholipid species in human plasma alone, and over 204 molecular SL species have been cataloged [95,96,99,100,101]. These SLs account for approximately 5% of the total amount of plasma lipids, wherein SMs represent 10% of total plasma PLs [95,96]. In addition, various SLs are present in serum, including S1P, dihydrosphingosine (dhSph), Cer, and SM [102]. Although the numbers of PL species in serum have not been specified, it is reasonable to assume that classes similar to those found in plasma exist due to the close physiological relationship between plasma and serum. Blood, being a highly dynamic system, interacts with all tissues and organs; thus, its metabolic components reflect overall health or disease states and indicate biological processes that occur in articular joints [58].

SF, crucial for joint lubrication and health, contains a wide array of PLs. Studies have identified 143 distinct PL and SL species in SF from patients with OA and RA [45,46], including PCs, LPCs, PEs, plasmalogens, PSs, PGs, SMs, Cer, hexosylceramides, dihexosylceramides, and minor glycerophospholipids [45,46]. Also, PI species and trace amounts of LPE have been found in all SF samples [103]. The composition and concentrations of these PLs can vary between healthy individuals and those with joint disorders, as discussed above [45,46,104].

PLs and SLs in SF are partially derived from synovial blood vessels and local production. Most PLs and SLs in SF, particularly PC, appear to diffuse from fenestrated blood capillaries near the synovial surface, wherein the pores of these capillaries are oriented primarily toward the synovial cavity [105]. As a result, SF can be regarded as an ultrafiltrate of plasma enriched with locally produced substances like cytokines, growth factors, and lubricants [64]. However, despite the dynamic nature of blood, the correlation between the levels of 168 metabolites in the blood and SF of patients with knee OA is modest [58]. Further, a portion of the lipids in SF can be stored in chondrocytes and in the extracellular matrix of articular cartilage [87,106,107].

### 6.2. Local Production

**Figure 1:**
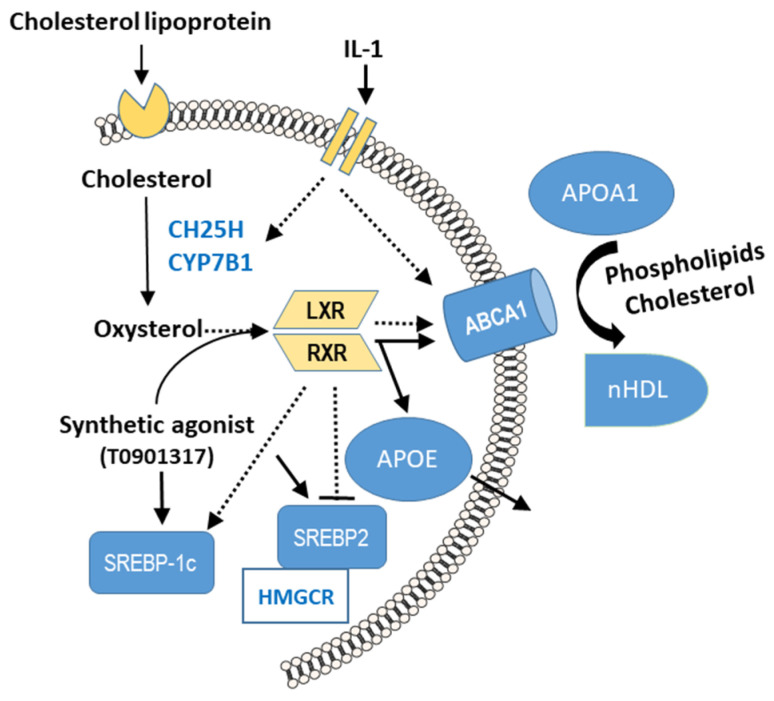
Proposed mechanism via which IL-1ß influences the release of phospholipids (PLs) from fibroblast-like synoviocytes (FLSs) during osteoarthritis (OA). IL-1ß upregulates cholesterol 25-hydroxylase (CH25H) and monooxygenase 25-hydroxycholesterol 7-alpha-hydroxylase (CYP7B1), leading to the accumulation of oxysterols, which act as natural ligands for nuclear liver X receptor (LXR). When oxysterols or synthetic agonists, like T0901317, activate LXR, the levels of active ATP-binding cassette transporter A1 (ABCA1) increase. ABCA1 facilitates the transport of PLs to extracellular apolipoprotein A1 (APOA1), promoting the formation of nascent HDL-c particles (nHDL). Also, oxysterols and T0901317 increase the expression of sterol regulatory element-binding protein 1c (SREBP1c), suggesting that enhanced fatty acid synthesis is required for PL production. Further, T0901317 upmodulates SREBP2, a key transcription factor that binds to the promoter region of 3-hydroxy-3-methylglutaryl-coA reductase (HMGCR)—the enzyme that controls the rate of cholesterol synthesis—thereby increasing its activity. Dotted line: IL-1; solid line: T0901317; arrow: stimulation; dash: inhibition. Reproduced with permission under an open access Creative Common CC BY license from [111].

Cells at the SF boundary, including those from articular cartilage, the synovium, and the meniscus, also contribute to the local production and release of lipids [108,109,110,111]. FLSs, recognized for their secretory functions, have been studied extensively (Figure 1). We have found that PL metabolism in OA joints is regulated intricately by cytokines, growth factors, and corticosteroids and that FLSs are crucial in modulating PL levels, which is essential for joint lubrication and protection against oxidative damage [108,109,110]:

In particular, IL-1β and tumor necrosis factor α (TNF-α) significantly influence the biosynthesis of PLs in cultured FLSs from OA knee joints [108]; specifically, IL-1β increases the production of PE and PE-based plasmalogens, which act as protective antioxidants. TNF-α stimulates the biosynthesis of all PL classes, whereas IL-6 has no significant effect. These findings indicate that FLSs adapt their PL composition in response to inflammatory stimuli during the progression of OA, perhaps contributing to the elevated PL levels that are observed in OA SF.

Transforming growth factor ß and insulin-like growth factor-1 significantly enhance the biosynthesis of PCs, SMs, and LPCs in cultured FLSs from human OA knee joints [109]. Bone morphogenetic proteins (BMPs) have weaker but specific effects, of which BMP-2 and BMP-7 upregulate PE and PE-based plasmalogens, which protect against reactive oxygen species. These growth factors may regulate the production of PL to support joint lubrication and potentially mitigate the progression of OA, highlighting their importance in maintaining joint health.

In contrast to stimulating the production of pulmonary surfactant, dexamethasone significantly inhibits the biosynthesis of PCs, PEs, PE-based plasmalogens, and SMs in cultured FLSs from human OA knees [110]. Our study showed that this suppressive effect is mediated by the glucocorticoid receptor, as evidenced by its reversal with the antagonist RU 486. Notably, adrenergic and cholinergic receptor agonists, which influence the synthesis of pulmonary surfactant, have no impact on PL biosynthesis in FLSs, reflecting distinct regulatory mechanisms between synovial joints and lungs [110].

Another recent study examined the function of IL-1β in stimulating the release of lubricating PLs from human OA FLSs, shedding light on potential compensatory mechanisms in joint lubrication during OA [111]. We found that IL-1β treatment significantly increases the release of PLs from FLSs by 1.4-fold compared with a control for IL-1ß (Figure 1). This effect was mediated through the upregulation of the cholesterol hydroxylases CH25H and CYP7B1, which produce oxysterols—natural agonists of liver X receptors (LXRs) [111]. These receptors, particularly LXRα, are pivotal in upregulating ATP-binding cassette transporter A1 (ABCA1), which facilitates the release of PLs from FLSs [111]. This study’s findings suggest a potential compensatory mechanism in OA joints, in which elevated PL levels offset the reductions in hyaluronan and lubricin concentrations.

### 6.3. Alterations in Lymphatic Vessels

In addition to diffusion from blood capillaries and local cellular contributions, alterations in lymphatic vessels can influence the composition of SF. Histochemical studies have shown that the articular lymphatic system, which transports macromolecules from the synovial space to draining lymph nodes, comprises fewer vessels in OA, possibly impairing lymphatic clearance and elevating macromolecular concentrations [112,113,114].

## 7. Functions

The literature on lipidomics and its physiological and pathophysiological significance is extensive, reporting increasingly improving analytical methods that quantify the lipidome of interest more accurately and comprehensively. In general, PLs and SLs are crucial components of cell membranes, forming a selective barrier that regulates the entry and exit of substances into and out of cells. They are vital in cell signaling and communication, serving as platforms for the attachment and activation of signaling proteins. These lipids play a role in numerous cellular processes, such as apoptosis, inflammation, proliferation, differentiation, and stress responses. PLs contribute to membrane fluidity and flexibility, whereas SLs form microdomains or “lipid rafts” that organize membrane proteins and influence signal transduction. Both types of lipids also participate in cellular trafficking and membrane remodeling and are precursors of bioactive signaling molecules. Given the breadth of the current state of the art of PLs and SLs, this review article will focus on the function of PLs and SLs in OA and provide an overview of our present state of knowledge.

### 7.1. Effects of PLs and SLs on Pain, Inflammation, and Destruction In Vivo

LPS induces a rapid and dose-dependent edematous reaction in rats [115]. This inflammatory response is mediated primarily by mast cell activation and subsequent histamine release, as evidenced by the 50% and 70% inhibition in this response that is achieved with the antihistamines chlorpheniramine and cyproheptadine, respectively [115]. The inflammatory effect varies between body regions, of which the rat paw shows greater sensitivity than the dorsal skin and pleural cavity [115]. Unlike other PLs, LPS has unique inflammatory properties based on its specific serine head group configuration. This structural feature enables LPS to activate mast cells and induce histamine release, leading to a potent inflammatory response. In contrast, LPCs, LPEs, and LPAs fail to elicit similar reactions [115]. Also, chemical modifications of the serine head group result in a loss of inflammatory activity, further highlighting the importance of this specific structure of LPS for its biological effects [115]. However, further pathways need to be investigated, and individual LPS species that could be involved in LPS-induced inflammation need to be identified. This study argues against nonspecific capillary damage; nevertheless, a thorough examination of the potential cytotoxic effects of LPS at higher concentrations is also warranted.

LPC 16:0 has emerged as a key mediator of chronic joint pain in rheumatic diseases, acting through acid-sensing ion channel 3 (ASIC3). A 2022 study by Jacquot et al. provided compelling evidence from human patients and mouse models, reporting that elevated LPC 16:0 levels in SF correlate with pain outcomes and that LPC 16:0-induced chronic pain behaviors are ASIC3-dependent [77]. This study examined two patient cohorts: 35 late-stage OA patients who were undergoing knee replacement surgery—with pain levels assessed on a visual analog scale and with the Knee Injury and Osteoarthritis Outcome Score—and 50 hospitalized patients with various rheumatic diseases (e.g., RA, psoriatic arthritis, gout) who donated SF. SF from 10 postmortem subjects without joint disease was used as a control group. Lipidomic analysis of SF samples was performed using high-resolution mass spectrometry to quantify nine LPC species. As a result, knee SF of the 35 OA patients had a 1.9-fold significantly higher concentration of total LPC compared with control specimens. LPC 16:0 was the most predominant species and the only one that increased significantly by 2.2-fold versus control samples [77]. This lipid species correlated with pain outcomes in OA patients, implicating it in chronic joint pain. Notably, LPC 16:0 was not specific to any particular joint disease—its levels were similar across various rheumatic conditions.

Using a rodent model, this group demonstrated that intraarticular injections of LPC 16:0 induced chronic joint pain and anxiety-like behaviors. These effects were mediated by ASIC3, which contributed to spinal sensitization and the persistence of pain [77]. The findings suggest that ASIC3 is crucial in mediating LPC-induced pain, consistent with previous research, but uniquely highlight its importance in chronic joint pain [116]. This study focused on LPC 16:0 and ASIC3, but other relevant LPC species and ion channels that are involved in nociceptive signaling pathways, such as TRPV1, have also been implicated [116,117,118].

Lysophosphatidic acid (LPA), a metabolite of LPC that is generated enzymatically by autotaxin, is a key factor in the joint damage and neuropathic pain that are associated with such diseases as OA and RA [80,119,120]. Recent research, including a study by McDougall and Reid [119], has demonstrated that intra-articular injection of LPA in rats affects joint degeneration and neuropathic pain, mimicking the characteristics of human joint diseases and indicating new potential therapeutic targets [119]. The degenerative changes included chondrocyte death, focal bone erosion, and synovitis [119]. The study found no significant differences in joint damage between male and female rats, suggesting that the effects of LPA on joint structure are independent of sex [119]. Previous studies have consistently linked LPA to neuropathic pain and joint degeneration, and McDougall and Reid’s findings align with these earlier observations, confirming that LPA contributes to nerve demyelination and altered firing patterns, which result in neuropathic pain [80,119,121]. Further, their research reinforces the function of LPA in synovial inflammation and cartilage damage, echoing findings that pharmacological inhibition of LPA receptors can mitigate these effects [80,120,121]

Recent work on OA has identified S1P as a potential therapeutic target; in particular, a study in mice demonstrated that inhibiting S1P signaling protects against cartilage degradation and reduces inflammation associated with OA [122]. In 2021, Cherifi et al. [122] found that osteoclast-derived S1P contributes to chondrocyte catabolism by upregulating MMP3 and MMP13 through S1P/S1P2 signaling. This process initiates the JNK signaling cascade, increasing matrix degradation in cartilage tissue. This study observed that mice that lack sphingosine kinase 1 (SphK1), an enzyme that phosphorylates sphingosine to S1P, in myeloid cells experience significantly less cartilage damage than control animals [122]. In addition, administration of the S1P2 receptor inhibitor JTE013 reversed osteoclast-mediated chondrocyte catabolism, whereas the S1P-neutralizing antibody sphingomab mitigated cartilage damage and synovial inflammation [122]. Moreover, SphK1-deficient mice expressed less MMP and underwent significantly less cartilage degradation, highlighting the potential of pharmacological and genetic approaches in addressing the pathology of OA [122].

However, the exclusive use of male mouse models might have failed to capture the entire complexity of OA in humans, potentially overlooking gender-specific differences in disease progression and treatment response [122]. Additionally, the study’s focus on a single pathway (S1P/S1P2) might have oversimplified the multifaceted pathogenesis of OA, which involves various tissues and mechanisms beyond cartilage degradation. In conclusion, the identification of S1P as a key factor in the progression of OA opens new avenues for therapeutic interventions. By demonstrating that the inhibition of S1P signaling protects against cartilage damage and reduces inflammation, Cherifi et al. [122] suggest a potential route for developing disease-modifying drugs for OA, a condition that lacks effective treatments, although further research and clinical trials would be needed to translate these findings to patients.

### 7.2. Effects of PLs and SLs on Cultured Fibroblast-like Synoviocytes

**Figure 2:**
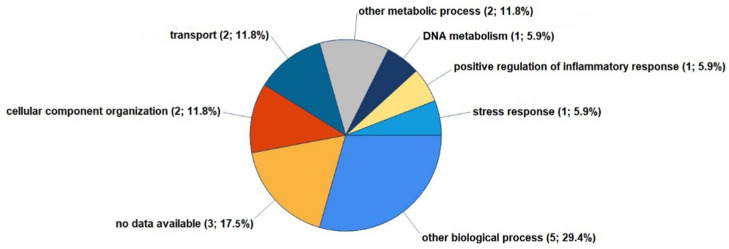
The biological processes of 8 proteins that were significantly regulated by LPC 16:0 in cultured FLSs. Using the Gene Ontology (GO) database, Proteome Discoverer 2.5 provided the GO slim categories for proteins reproducibly upregulated at least 1.2-fold or downregulated 0.8-fold in FLSs treated with LPC 16:0 for 48 h. Reproduced with permission under an open access Creative Common CC BY license from [123].

Using a novel proteomic MS analytical method, two recent studies by Timm et al. explored the role of PL and SL species in modulating protein expression in cultured FLSs from OA joints [123,124]. Our publication in 2023 focused on LPC species and their metabolites LPA and found that small chemical variations in LPCs significantly affect protein and PL synthesis, especially in advanced stages of OA characterized by low IL-1ß levels [123]. LPC 16:0, in particular, significantly inhibits PL release and synthesis and affects the expression of eight proteins (Figure 2). For instance, promising proteins such as CD81 antigen, calumenin, and B4E2C1 were identified as potential modulators of inflammatory and catabolic processes, implicating them as new therapeutic targets in OA [123]. Notably, LPC species have been reported to bind to such receptors as the G-protein-coupled receptors GPR132 (G2A) and GPR4 and Toll-like receptors 2 and 4 in other cells. On stimulation by LPC, various signaling pathways are activated, including NF-κB, p38 MAPK, and JNK, which can influence several functions, such as the release of inflammatory factors, oxidative stress, and apoptosis [125,126].

**Figure 3:**
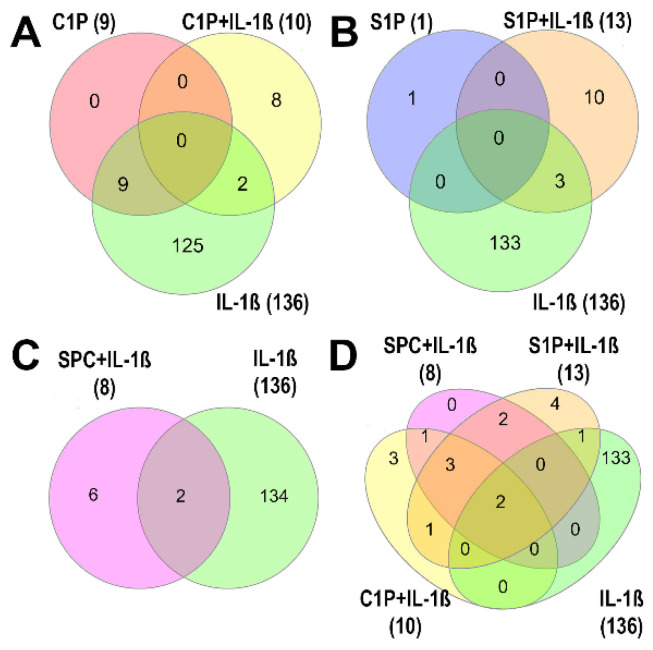
Venn diagram illustrating the overlap of proteins from cultured FLS consistently modulated by (**A**) C1P (red), C1P in the presence of IL-1ß (yellow), and IL-1ß alone (green); (**B**) S1P (blue), S1P in the presence of IL-1ß (orange), and IL-1ß alone (green); (**C**) SPC in the presence of IL-1ß (violet) and IL-1ß alone (green); and (**D**) IL-1ß in the presence of C1P (yellow), S1P (orange), or SPC (violet). The number of reproducibly regulated proteins is given in parentheses. Reproduced with permission under an open access Creative Common CC BY license from [124].

The second of the two studies by Timm et al., published in 2024, complemented the poorly understood pathophysiological function of the three major SLs—ceramide-1-phosphate (C1P), S1P, and sphingosylphosphorylcholine (SPC)—in cultured FLSs from human OA knees [124]. C1P is a low-affinity agonist that may bind to a Gi protein-coupled receptor in macrophages, but it is unknown whether such receptors exist in articular joint cells [127]. S1P binds strongly to G-protein-coupled S1P receptors (S1PR1 to S1PR5), with various effects across tissues [128]. S1PR1 is found in synovial lining cells, vascular endothelial cells, and inflammatory mononuclear cells of the synovium during RA and OA [129]. SPC, structurally similar to S1P, can act as a low-affinity agonist for S1P receptors [130] or as a second messenger for intracellular calcium release [131]. In the study by Timm et al. (2024), C1P proved to be the most influential lipid, as it significantly modulated nine proteins, while S1P influenced only one protein; SPC had no significant single effect (Figure 3). Notably, combining the three SLs with IL-1β enhanced their regulatory effects on protein expression [124]. Stimulation with C1P had partially opposing effects, wherein proinflammatory, anti-inflammatory, and catabolic proteins were upregulated, in addition to other proteins. For instance, C1P modulated such proteins as metallothioneins-1F, ferritin, charged multivesicular body protein 1b, and metal cation symporter ZIP14, which are involved in inflammatory, anabolic, catabolic, and apoptotic processes. Our study identified several proteins that are implicated in the pathogenesis of OA throughout disease progression, although their specific functions have yet to be validated.

These studies by Timm et al. provide intriguing insights into the function of lipid species in OA [123,124]. However, our studies were conducted in vitro, which might have failed to capture the complex in vivo interactions within OA joints. Future studies should investigate stage-specific therapeutic interventions targeting LPC signaling pathways, as these play a role in advanced OA stages characterized by low IL-1β levels. Determining the mechanisms through which C1P influences inflammatory and apoptotic processes could yield valuable insights. Further, research on lipid-specific pathways and their interactions with cytokines like IL-1β could elucidate the complex regulatory mechanisms that drive OA, potentially identifying novel diagnostic and therapeutic strategies.

PS, a PL that is found in cell membranes, has been shown to have promising anti-inflammatory and analgesic effects in RA models, suggesting its potential therapeutic application for inflammatory joint diseases, including OA [132]. PS significantly inhibits the production of key inflammatory mediators, including IL-6, IL-8, prostaglandin E2 (PGE2), and vascular endothelial growth factor (VEGF), in IL-1β-stimulated FLSs from RA patients [132]. This inhibition was accompanied by the suppression of p38 and c-Jun N-terminal kinase phosphorylation, as well as the abrogation of IκBα phosphorylation, which is crucial for NF-κB-mediated inflammatory responses. In in vivo experiments that used rat models of carrageenan-induced arthritis and hyperalgesia, PS had antiarthritic and analgesic effects. Oral administration of PS reduced paw swelling, improved the weight distribution ratio, and decreased pain responses in these models, showing comparable efficacy as prednisolone in certain cases [132]. These findings suggest that PS has potential as a therapeutic agent or dietary supplement for managing inflammatory joint diseases, constituting a promising area of further research and development in rheumatology. However, additional studies are needed to provide information on its bioavailability and pharmacokinetics when administered orally. Whereas the study above focused primarily on RA, its findings suggest potential applications for the treatment of OA. The anti-inflammatory properties of PS, particularly its inhibition of IL-6, IL-8, VEGF, and PGE2, could be beneficial in mitigating inflammation and pain in OA joints. The ability of PS to inhibit the JNK and p38 MAPK pathways may also slow disease progression in OA. Future research should provide data on the effects of PS on MMPs and ADAMTs, which are critical in the degradation of cartilage in OA.

### 7.3. Effects of PLs and SLs on Cultured Chondrocytes

S1P, a bioactive lipid mediator, has emerged as a potential therapeutic target in OA due to its anti-inflammatory and cartilage-protective properties. S1P counteracts IL-1β-induced inflammation and cartilage degradation by modulating specific receptors, particularly S1P_2_ and S1P_1_, suggesting that it mitigates OA progression and preserves joint health [133,134,135]. Human and bovine chondrocytes express the S1P receptors S1P_1_, S1P_2_, and S1P_3_ but not S1P_4_ or S1P_5_ [133,135]. S1P_2_ receptors were identified as the most prevalent subtype in human OA cartilage and chondrocytes in vitro [134], and S1P_1_ is involved in S1P-dependent inhibition of IL-1ß-induced effects in human chondrocytes [135]. This receptor specificity is crucial in mediating the protective effects of S1P against inflammatory cytokines, particularly in counteracting the IL-1ß-induced activation of NF-κB p65 and phosphorylation of p38 MAPK [134,135]. S1P dose-dependently inhibits the expression of key inflammatory mediators and catabolic enzymes, such as inducible nitric oxide synthase, cyclooxygenase-2 (COX-2), MMP-1, MMP-3, MMP-13, MMP-14, and ADAMTS-4 [133,134,135]. These effects decrease PGE2 synthesis and glycosaminoglycan (GAG) degradation in human cartilage explants [133,134,135]. The anti-catabolic action of S1P is mediated through S1P_2_, as demonstrated by pharmacological inhibition and siRNA knockdown experiments [134]. In addition, the anti-inflammatory action of S1P, mediated by the inhibition of IL-1ß-induced COX-2 expression, was blocked by the S1P_1_ receptor antagonist W146 [135].

S1P influences chondrocyte proliferation, angiogenesis, and inflammation in articular cartilage, suggesting that it has destructive and potentially regenerative effects in such conditions as OA and RA. Masuko et al. (2007) have shown that S1P stimulates PGE2 production in human articular chondrocytes by activating COX-2 and MAPKs [136]. This increase in PGE2 is associated with the downregulation of proteoglycan aggrecan, a crucial component of cartilage matrix [136]. These findings [136] thus contradict those of Moon et al. [135], who reported inhibition of COX-2 activity and thus lower synthesis of PGE2, whereas Stradner et al. [133] observed no effect of S1P on IL-1-induced COX-2 expression. The reduced catabolism in chondrocytes and the attenuation of OA in mice after S1P inhibition, as described by Cherifi et al. [122], cannot be reconciled with the anti-catabolic and anti-inflammatory effects of S1P described above [133,134,135]. Further research is needed to resolve these discrepancies. Also, S1P enhances VEGF expression in human chondrocytes, potentially promoting angiogenesis within arthritic cartilage [137]. In primary rat chondrocytes, S1P stimulates cellular proliferation through ERK activation, suggesting involvement in cartilage repair processes [138].

LPA has been identified as a promising signaling molecule with significant implications for cartilage health and regeneration. In vitro studies have highlighted its multifaceted role in promoting chondrocyte proliferation, increasing extracellular matrix organization, and stimulating angiogenesis. Several key findings have been reported regarding LPA, noting the following:

- It enhances the angiogenic capacity of human chondrocytes by upregulating factors such as VEGF, IL-8, and MMP-9 through Gi/NF-κB signaling [139].
- It improves the tensile properties of engineered fibrocartilage by inducing cytoskeletal reorganization and enhancing extracellular matrix alignment [140].
- It stimulates rat primary chondrocyte proliferation via ERK activation, mediated by Gi/o protein-coupled receptors [141].
- As part of the autotaxin-LPA1 axis, it regulates chondrocyte proliferation and cartilage formation by promoting S-phase entry through the assembly of integrin-mediated fibronectin [142].

These findings highlight the potential of LPA in tissue engineering, cartilage repair, and developmental biology, demonstrating its function in cellular proliferation, matrix organization, and angiogenesis across experimental models. LPA has consistently demonstrated its ability to promote cell proliferation in various models, including human and rat chondrocytes, as well as engineered tissues [139,140,141]. Gi protein-coupled receptor signaling has arisen as a central mechanism, the downstream pathways of which—such as NF-κB, ERK, and integrin-mediated adhesion—are crucial in mediating the effects of LPA [139,141,142]. Also, its involvement in extracellular matrix remodeling has been highlighted, particularly with regard to its capacity to enhance extracellular matrix organization, which is critical for improving mechanical properties and cell adhesion in engineered tissues [140,142].

### 7.4. Role of PLs in Ferroptosis During OA

Ferroptosis, an iron-dependent form of cell death that is characterized by lipid peroxidation, is an important factor in the progression of OA, and the interested reader is referred to external articles, including the recent review [143]. Briefly, this process, driven by iron accumulation and the oxidation of PLs in chondrocytes, contributes to cartilage degradation and inflammation, unveiling potential therapeutic strategies for managing this prevalent joint disease [143]. Polyunsaturated fatty acid (PUFA)-containing PLs, particularly PE, are key substrates for lipid peroxidation in ferroptosis during OA. This process is facilitated by enzymes like lipoxygenases and Acyl-CoA synthetase long-chain family member 4 [143]. The resulting accumulation of lipid peroxides and reactive oxygen species drives chondrocyte death and cartilage degradation [143,144].

### 7.5. Glycosphingolipids in OA and Cartilage Regeneration

Glycosphingolipids, especially gangliosides, are involved in the pathogenesis of OA and cartilage repair. Glycosphingolipids are key components of cell membranes, which comprise a hydrophobic ceramide and a hydrophilic oligosaccharide residue. A review by Homan et al. (2024) highlights their potential as biomarkers and therapeutic targets, with promising applications in cell-based regeneration strategies and future multifactorial treatments [145]. Briefly, in OA cartilage, a notable decrease in major gangliosides compromises tissue homeostasis and resilience to mechanical stress. Gangliosides regulate signaling cascades—including the MAPK pathway—that influence chondrocyte differentiation, cartilage metabolism, and apoptosis. Their depletion exacerbates cartilage degradation by increasing the levels of inflammatory cytokines and MMPs. The therapeutic potential of gangliosides lies in their ability to protect cartilage from degeneration and promote repair when supplemented or restored. Studies suggest that replenishing specific gangliosides enhances the resilience and regeneration of cartilage [145]. Exogenous administration of specific gangliosides can facilitate cartilage repair processes through the following mechanisms:

- Promoting chondrogenic differentiation of mesenchymal stem cells
- Reducing inflammatory responses in OA cartilage
- Modulating signaling pathways that influence cartilage homeostasis, such as MAPK.

The therapeutic application of gangliosides in cartilage repair strategies is an emerging discipline, wherein studies have suggested that their incorporation in biomaterials or cell-based therapies could significantly improve outcomes of treating cartilage injuries and OA [145].

### 7.6. PLs and Joint Lubrication

**Figure 4:**
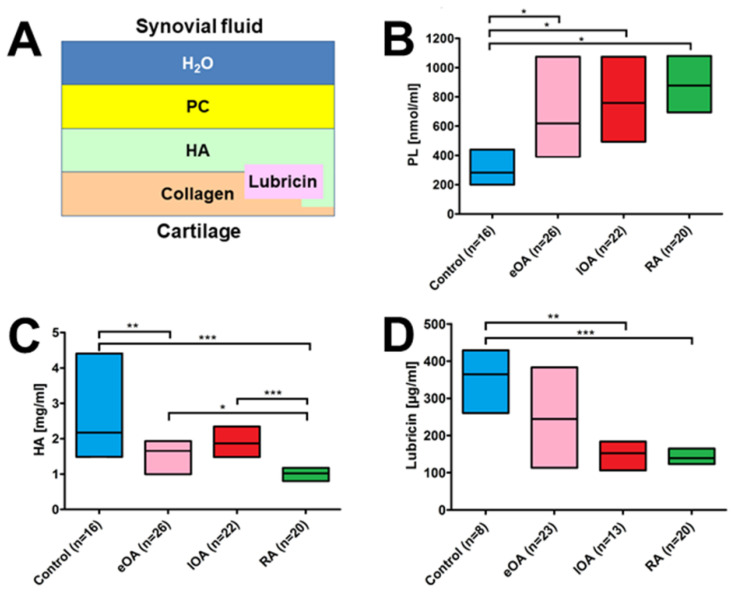
Boundary layers on the surface of articular cartilage. (**A**) Schematic representation of the proposed structure of an interface with lubricin molecules (purple) located in the outermost superficial zone (brown) of the cartilage, as previously reported [147,150]. These lubricin molecules anchor hyaluronic acid (HA, green) on the cartilage surface. HA forms a complex with phospholipids (PL, yellow) like phosphatidylcholine (PC). The outer, highly hydrated (blue) phosphocholine head groups of, e.g., PC, minimize friction through a hydration lubrication mechanism, as described in the text. The levels of (**B**) PLs, (**C**) HA, and (**D**) lubricin were quantified in synovial fluid collected from postmortem controls, as well as from patients with early osteoarthritis (eOA), late osteoarthritis (lOA), and rheumatoid arthritis (RA). *p*-values less than 0.05 were considered statistically significant: * 0.01 < *p* ≤ 0.05, ** 0.001 < *p* ≤ 0.01, *** *p* ≤ 0.001. Figure 4B–D are reproduced with permission under the Creative Commons Attribution License from [64], © 2015 Kosinska et al.

PLs and SLs are crucial in lubricating articular cartilage through the formation of highly effective boundary layers. These lipids, abundant in synovial joints, exploit the hydration lubrication mechanism to reduce friction under physiological conditions [146,147,148,149,150]. By forming robust, hydrated bilayers at the cartilage surface, they affect smooth sliding between opposing surfaces, resulting in extremely low friction coefficients, even under the high pressures that are typical of joint articulation. Recent research has highlighted the synergistic effects of lipid mixtures in enhancing these lubrication properties, of which certain combinations provide superior resistance to breakdown under mechanical stress [146]. This interplay between lipid types, paralleled by their interactions with other SF components, such as hyaluronic acid (HA) and lubricin, creates a complex yet highly efficient lubrication system that is essential for maintaining joint health and function (Figure 4) [146,147,148,149,150].

Hydration lubrication is a key element of the exceptional lubricating properties of lipid bilayers on articular cartilage surfaces (Figure 4A). This mechanism relies on the highly hydrated phosphocholine headgroups of PLs and SM, which bind water molecules tightly [146,148,149]. The resulting hydration shells resist compression and allow gliding between opposing surfaces, yielding friction coefficients as low as μ ≈ 10^−4^, even under physiological pressures of 5 MPa or more [146]. The tightly bound water molecules around the lipid headgroups provide a slip plane that maintains low friction while resisting squeeze-out under load, rendering this mechanism particularly effective for joint lubrication.

PCs and SMs form robust boundary layers in lubricating articular cartilage. Specific PCs, like DPPC and POPC, are extremely effective due to their zwitterionic phosphocholine headgroups [146]. SM lipids contribute to bilayer stability based on their saturated acyl chains and ability to form gel-phase domains [146]. A 5-component mixture of DPPC, POPC, dioleoylphosphatidylcholine, 1-palmitoyl-2-oleoyl-sn-glycero-3-phosphoethanolamine, and egg SM has been shown to have superior lubrication properties by balancing liquid-phase lipids for lubrication and gel-phase lipids for mechanical stability [146]. These lipid mixtures exhibit superior lubrication properties compared with single-component systems, demonstrating a clear synergistic effect [146,149].

Interactions between PL bilayers and other SF components create a synergistic lubrication system (Figure 4A). HA forms complexes with PCs through hydrophilic and hydrophobic forces, exposing hydrated phosphocholine groups [147,148,149]. Lubricin or proteoglycan 4 immobilizes HA at the cartilage surface, facilitating the attachment of PLs and forming stable boundary layers [147,148,149]. This system maintains robust, low-friction surfaces under physiological stresses [147,148,149,150].

A recent study revealed that degradation of key lubricating molecules in SF significantly impacts chondrocyte health and cartilage mechanics following joint injury [151]. Specifically, the removal of HA and lubricin from SF led to increased middle zone chondrocyte damage and higher shear strain loading magnitudes. This alteration in SF composition also changed chondrocyte sensitivity to mechanical loading, suggesting a direct link between lubrication quality and cellular response to stress [151]. These findings highlight the critical role of SF lubrication in maintaining joint health and preventing post-traumatic OA. The research underscores the potential for targeted therapies aimed at preserving or restoring SF composition as a strategy for mitigating post-traumatic OA development following joint injury [151,152].

Kosinska et al. (2015) provided crucial quantitative data on SF components in healthy and diseased joints, complementing the reported data above on articular cartilage lubrication mechanisms [64]. Our study found that HA and lubricin levels decrease in OA and RA, PL concentrations increase in diseased SF, and the molecular weight distribution of HA shifts toward the lower ranges in OA and RA (Figure 4B-D) [64]. The resulting detailed profile of SF components at various stages of OA and RA allowed us to gain a nuanced understanding of how alterations in lubricants may contribute to the pathogenesis and progression of joint diseases. This study also monitored changes in fatty acid saturation and the chain lengths of PLs in OA and RA [64]. These changes could affect the ability of the zwitterionic head groups of PLs to interact with water molecules, potentially impacting their role in reducing friction at cartilage surfaces under high pressure by hydration lubrication [146,147,148,149]. These quantitative data on molecular weight shifts and compositional changes in lubricants during disease progression could guide the formulation of more effective viscosupplementation therapies or synthetic boundary lubricants to combat OA and RA.

## 8. Potential Clinical Application

### 8.1. PLs and SLs as Biomarkers

Recent work has identified a significant link between lipid metabolism and the progression of OA, hinting at potential early diagnostic tools and targeted therapies. PL and SL levels in SF, serum, or plasma are elevated in early-stage and/or late-stage OA patients compared with healthy controls [45,46,48,50,51,52,55,57,60], suggesting that lipid markers are indicators of the early stages of OA, even before radiological detection is possible. In this context, a lipidomic study by Surowiec et al. (2016) is noteworthy, finding that pre-symptomatic RA patients have higher plasma levels of PLs and SMs than controls [69]. LPCs, in particular, were significantly higher in individuals before the onset of RA, indicating their potential as early biomarkers of RA [69].

The lipid profile changes that are observed during the progression of OA are not limited to PLs and SLs. For instance, a lipidomic study of SF fatty acids noted differences between OA and control subjects, including a tendency toward lower levels of omega-6 fatty acids in OA [85]. This alteration in fatty acid composition could contribute to the inflammatory environment that is characteristic of OA. Moreover, the association between lipid markers and OA extends beyond their diagnostic potential. A study that examined the NHANES database by Mendelian randomization analysis found that LDL cholesterol is actually a protective factor for OA, whereas the function of HDL cholesterol remains unknown, requiring further investigation [86]. The identification of specific lipid biomarkers that are associated with OA creates new possibilities for monitoring disease development and determining treatment efficacy.

For instance, certain choline-containing lipid classes and molecular species have been identified as biomarkers of chronic joint pain, regardless of the underlying pathology [50]. This finding suggests that lipid markers are useful for not only tracking structural changes in OA but also assessing symptomatic progression. As our understanding of lipid metabolism in OA deepens, it becomes increasingly clear that these markers are crucial in disease progression. The ability to follow these changes using noninvasive blood tests could allow for earlier intervention and more personalized treatment strategies that are based on individual lipid profiles.

The potential for making an early diagnosis using lipid biomarkers is supported by genetic studies. Recent bioinformatic analyses have identified several genes that correlate with lipid metabolism in OA, including hub genes that correspond to proinflammatory cytokines and immune-related cells in peripheral blood [62]. These genetic markers could complement lipid biomarkers, providing a more comprehensive approach to diagnosing OA earlier. As research in this area progresses, the integration of lipid biomarker analysis with other diagnostic tools, such as imaging techniques and clinical assessments, could lead to more accurate and earlier detection of OA. This multimodal approach could significantly improve patient outcomes by enabling targeted interventions before substantial joint damage occurs.

### 8.2. Future OA Treatment Strategies

Our evolving understanding of the role of lipid metabolism in the pathogenesis of OA is paving the way for innovative treatment strategies that target specific lipid pathways. These approaches aim to address the metabolic and inflammatory aspects of OA, potentially constituting more effective interventions than current therapies. One promising avenue involves modulating the activity of specific PL and SL species that are elevated in OA patients. By targeting the transporters and enzymes that mediate their synthesis or breakdown, it may be possible to normalize lipid profiles and mitigate their proinflammatory effects. For instance, inhibitors of sphingosine kinase, an enzyme that is involved in SL metabolism, have shown potential in reducing inflammation in preclinical models of arthritis [153,154,155].

The discovery that S1P is involved in the progression of OA reveals new therapeutic possibilities. The detection of S1P in OA human tissue is perhaps a translatable finding—from in vitro and mouse studies to patient care [122,133,134,135,136,137,138]. However, the available in vitro and in vivo findings are partly contradictory. The efficacy of the S1P1 receptor agonist in vitro [135] and the in vivo application of antibodies that target S1P and drugs that block the S1P2 receptor in protecting cartilage and reducing joint inflammation in mice represent an encouraging path toward the development of treatments that could alter the course of OA, which current therapies fail to do [122].

Given that S1P activates MAPK pathways (such as ERK and p38), future therapeutic strategies could involve modulating these pathways to achieve the desired effects on cartilage metabolism and inflammation [136,137,138]. In addition, shaping the effects of S1P on chondrocyte proliferation could aid cartilage repair and regeneration in OA [138]. The regulation of VEGF expression by S1P presents opportunities for managing angiogenesis within joints, which could be beneficial in certain stages of disease progression [137].

Lipid-based therapies could also focus on enhancing the production of anti-inflammatory lipid mediators. Supplementation with omega-3 fatty acid, for example, has shown potential in reducing OA-related pain and inflammation by promoting the synthesis of specialized pro-resolving mediators [84,156]. Future treatments might involve the targeted delivery of pro-resolving mediators or their precursors to affected joints, perhaps eliciting a more direct and potent anti-inflammatory effect.

The identification of LPC species as stimulators of inflammatory responses in articular FLSs represents another potential therapeutic target [77,123]. The development of antagonists for LPC receptors or inhibitors of LPC synthesis could diminish the inflammatory cascade in OA joints. This approach could be particularly beneficial in early-stage OA, in which such interventions might prevent the establishment of chronic inflammation.

Targeting the autotaxin-LPA pathway is another promising option for treating joint diseases and the associated neuropathic pain. LPA receptor antagonists, such as Ki16425, have demonstrated efficacy in reducing synovial inflammation, cartilage damage, and bone erosion in preclinical models [80,120,121].

With regard to tissue engineering, LPA could increase the mechanical strength of engineered constructs, particularly for avascular tissues, such as the knee meniscus [139]. LPA has the potential to repair cartilage and treat OA based on its ability to stimulate chondrocyte proliferation and protect against apoptosis [140,142]. Its angiogenic properties may aid in bone regeneration and fracture healing [139]. Additionally, targeting the autotaxin-LPA pathway could address congenital skeletal disorders or growth deficiencies [142]. However, careful consideration of the risks, such as uncontrolled angiogenesis and hypertrophic differentiation of chondrocytes, and dosing strategies will be needed before clinical implementation [139,141].

Multifactorial approaches that combine glycosphingolipid supplementation with novel cell-based therapies could be a promising future advance in OA treatments [145]. However, clinical trials should be performed to translate the experimental findings to date into viable treatments, focusing on evaluating the efficacy and safety of glycosphingolipid-based therapies in humans.

Viscosupplementation, which entails the injection of HA into affected joints, aims to restore the viscosity of SF and improve joint lubrication, potentially alleviating symptoms and slowing disease progression. Altered levels of lubricin, PLs, and SLs, as reported by Kosinska et al. (2015), represent opportunities for developing novel synthetic or bio-inspired lubricants [64,157]. Thus, combination therapies that target multiple pathways might be more effective than single-agent treatments. Such therapies could involve combining HA supplementation with compounds that stabilize lubricin or modulate PL metabolism [64,157]. In addition, these research directions could influence the design of joint prostheses by promoting the use of PL-like materials or coatings that mimic natural lubrication mechanisms and improve the performance and longevity of prostheses.

Emerging insights into the variability of the composition of blood or SF between patients with OA and healthy controls could guide the development of personalized treatment strategies. By analyzing individual blood or SF profiles, clinicians could tailor interventions to address specific biochemical imbalances, potentially optimizing therapeutic outcomes. This approach aligns with the growing trend toward precision medicine in rheumatology, in which treatments are customized to individual patient characteristics. Future research could focus on developing rapid diagnostic tools to assess the composition of serum, plasma, or even SF at the point of care, enabling more targeted and effective management of joint diseases.

Gene therapy approaches that target lipid metabolism pathways are also being explored. With the identification of hub genes that are related to lipid metabolism in OA, such as those that correspond to proinflammatory cytokines and immune-related cells, RNA interference and CRISPR-based techniques could be applied to modulate their expression [62]. This genetic approach could become a more precise method of regulating lipid metabolism in OA.

The systemic nature of lipid profile changes in OA suggests that whole-body metabolic interventions could be beneficial [86,158]. Lifestyle modifications, including dietary changes and exercise regimens that are tailored to improve lipid profiles, may complement pharmacological treatments. Personalized nutrition plans that are based on individual lipidomic profiles could become a standard component of the management of OA. Drugs that target lipid metabolism pathways, such as statins and PCSK9 inhibitors, may reduce systemic inflammation and oxidative stress, thereby slowing cartilage degradation [159,160,161,162,163]. Future treatments might also involve carefully modulating LDL levels to achieve a balance that supports joint health without increasing cardiovascular risk.

In summary, the development of lipid-targeted therapies represents a paradigm shift in the treatment of OA, redirecting such efforts from managing symptoms toward addressing the underlying metabolic dysfunction. As these strategies advance through preclinical and clinical trials, they will hold increasingly greater promise of becoming more effective, personalized treatments that could significantly improve outcomes for OA patients.

## 9. Conclusions

OA is increasingly being linked to altered lipid metabolism, with recent research highlighting the crucial roles of PLs and SLs in its pathogenesis. These lipids are promising biomarkers of the progression of OA and potential targets for innovative therapeutic approaches. Studies have identified higher levels of specific PLs and SLs in the SF and blood of OA patients compared with controls. These changes are stage-dependent, wherein elevated concentrations are observed already in the early stages of OA, implicating them as potential biomarkers of disease severity. Local lipid production by FLS in the joints adapts in response to inflammatory stimuli and contributes to the elevated PL levels in OA SF, with PLs originating mainly from the blood capillaries. This phenomenon underscores the interplay between local cellular activity and systemic lipid metabolism in OA pathology. Animal models, such as dogs and mice, exhibit lipidomic profiles that are similar to those in humans during OA progression, rendering them valuable for studying lipid-related mechanisms. However, species-specific differences will necessitate careful translation of the findings to clinical applications.

Targeting lipid pathways is a novel therapeutic avenue. Promising strategies include modulating lipid species like S1P, LPC, and LPA, as well as leveraging anti-inflammatory lipid mediators, gene therapy, and precision medicine approaches to normalize lipid profiles, reduce inflammation, and promote cartilage repair. These potential advancements, combined with lifestyle interventions and novel biomaterials for joint lubrication, would represent a shift toward multifactorial therapies to address OA’s underlying metabolic dysfunction. Overall, future studies need to exploit the potential of PLs and SLs not only as biomarkers of the early stages of OA but also as therapeutic targets for OA by developing novel joint lubricants, investigating lipid-modulating interventions, and validating these approaches in animal models to ultimately develop new and even personalized treatment strategies.

## Figures and Tables

**Table 1:** Altered PL and SL levels in human body fluids in patients with common diseases.

Disease	Lipid Type	Fluids/Tissues Investigated	Authors
Alzheimer’s Disease	PLs ↓, SLs ↑↓	Cerebrospinal fluid	[21,22,23]
Antiphospholipid Syndrome	PLs	Blood	[24,25]
Atherosclerosis	PLs ↑, SLs ↑	Plasma, Plaque	[26,27]
Atopic Dermatitis	PLs ↓, SLs ↑↓	Skin	[28,29]
Chronic Kidney Disease	PLs ↑, SLs ↑	Serum, Plasma	[30,31]
Type 2 diabetes	SLs ↑↓	Plasma	[32,33]
Metabolic Syndrome	SLs ↑↓	Serum, Plasma	[33,34,35]
Multiple Sclerosis	PLs ↑↓, SLs ↑↓	PBMC, WBC, Serum, Plasma, Brain tissue, Cerebrospinal fluid,	[36,37,38,39]
Nonalcoholic Fatty Liver Disease	PLs ↑↓, SLs ↑↓	Plasma, Liver tissue	[40,41,42]
Obesity	SLs ↑↓	Serum, Plasma, Artery, Heart, Liver, Muscle	[43,44]
Osteoarthritis	PLs ↑↓, SLs ↑	Serum, Plasma, Synovial fluid, Synovial tissue	[45,46,47,48,49,50,51,52,53,54,55,56,57,58,59,60,61,62,63,64]
Parkinson’s Disease	PLs ↑↓, SLs ↑↓	Serum, Plasma, Cerebrospinal fluid	[65,66,67]
Rheumatoid Arthritis	PLs ↑↓, SLs ↑	Serum, Plasma, Urine Synovial fluid	[45,46,50,57,64,68,69,70,71]

PBMC: Peripheral Blood Mononuclear Cells; WBC: White Blood Cells.

**Table 2:** Lipidomic studies in synovial fluid, serum, or plasma obtained from OA patients and controls.

Study/Year	Patients [Number/Disease Stage]	Focus Area	Methods	Key Findings
Castro-Perez et al., 2010 [51]	18 early OA; 15 mod. OA; 26 controls	Lipidomic analysis in plasma of OA patients vs. controls	UPLC-TOF-MS	Significant separation between controls, early OA, and moderate OA groups. The 284 identified fatty acids, lipids, and triglycerides included 186 PL species (65 PCs, 43 SMs, 40 PSs, 22 LPCs, 7 PIs, 4 PEs, 3 PGs, 2 LPEs).
Kosinska et al., 2013 [45]	17 early OA; 13 late OA; 9 controls	Lipidomic analysis of PLs in SF of OA patients vs. controls	ESI-MS/MS	Elevated levels of a range of 130 PL species across 8 classes, including PCs, LPCs, PE, and PE P in SF of early and late-stage OA patients compared to controls. For instance, PC concentrations were 2.7 times higher in early OA and 5.4 times higher in late OA compared to controls.
Kosinska et al., 2014 [46]	17 early OA; 13 late OA; 9 controls	Lipidomic analysis of SLs in SF of OA patients vs. controls	LC-MS/MS, ESI-MS/MS	Elevated SM and Cer levels in OA SF compared to controls: SM levels were elevated 2.4-fold in early and 4.4-fold in late OA; Cer levels increased 2.0-fold in early and 3.9-fold in late OA.
Zhang et al., 2014 [47]	80 late OA	Metabolomic profiling of SF for phenotyping	Metabolomics using the MS-based kit AbsoluteIDQ™ p180	Revealed two main metabolically distinct phenotypes in OA patients. PLs played a crucial role in distinguishing between OA subgroups.
Zhang et al., 2015 [58]	69 late OA	Ratio of paired plasma to SF metabolite levels	Metabolomics using the MS-based kit AbsoluteIDQ™ p180	Revealed only modest correlations between plasma and SF metabolite concentrations in OA patients. Metabolite ratios demonstrated stronger correlations, particularly those involving glycerophospholipids (ρ = 0.6–0.8).
Zhang et al., 2016 [59]	126 late OA; 101 controls	Metabolomic profiling of plasma and SF	Metabolomics using the MS-based kit AbsoluteIDQ™ p180	Revealed association between altered PC metabolism with both OA and type 2 diabetes mellitus, with specific metabolites such as PC O-34:3 and PC O-36:3 identified as potential biomarkers of their concurrence.
Zhang et al., 2016 [52]	136 late OA; 121 controls	LPC-to-PC ratio in plasma; correlation with need for TKR	Metabolomics using the MS-based kit AbsoluteIDQ™ p180	Advanced OA requiring total knee replacement (TKR), with patients having a ratio ≥ 0.09 being 2.3 times more likely to undergo TKR over a 10-year period. The ratio may be used as a biomarker for risk stratification.
Zhai et al., 2019 [54]	158 mod. OA	LPC-to-PC ratio in serum; correlation with symptoms	Metabolomics using the MS-based kit AbsoluteIDQ™ p180	Knee OA patients with a ratio ≥ 0.088 were 2.93 times more likely to respond positively to anti-inflammatory treatment. Demonstrates potential for personalized treatment approaches.
Zhai et al., 2019 [53]	139 mod. OA	LPC-to-PC ratio in serum; correlation with cartilage loss	Metabolomics using the MS-based kit AbsoluteIDQ™ p180	Revealed association of LPC 18:2/PC 44:3 ratio with cartilage volume loss over time, particularly in the lateral compartment, as measured by MRI. Biomarker may enable monitoring disease progression.
Tootsi et al., 2020 [55]	70 late OA; 82 controls	Metabolomic profile of serum from late OA vs. controls	Metabolomics using the MS-based kit AbsoluteIDQ™ p180	Revealed alterations in amino acid, biogenic amine, and lipid profiles in serum of late-stage OA patients versus controls. LPC 20:4 and SM 20:2 were elevated and 8 PC species were decreased in serum of OA patients.
Rocha et al., 2021 [48]	7 early OA; 8 late OA; 4 controls	Lipidomic profile in SF; correlation with cartilage damage	Targeted phospho-lipidomic analysis using MRM/MS	Higher PC levels in early-stage OA SF vs. late-stage OA; stratification of early-stage OA into two subgroups based on PL profiles.
Bocsa et al., 2022 [49]	12 early OA; 19 late OA	Metabolomic profiling of SF in knee OA	Untargeted metabolomics using LC-MS	Identified 4 PLs with stage-dependent differences between early- and late-stage OA; Cer (d18:1/20:0) was higher in early-stage OA, while most other metabolites were enriched in late-stage OA patients.
Loef et al., 2022 [56]	216 OA	Plasma lipidomics and OA severity	Lipidyzer™ platform, LC-MS/MS	Minor lipidomic differences between various OA stages; limited association between lipid profiles and radiographic severity of OA.
Khoury et al., 2023 [50]	18 OA; 10 controls	Choline-containing lipids in SF as biomarkers	High-resolution MS with ESI-MS/MS	LPC 16:0, LPC 18:0, and both PC and PC P class elevated in SF of OA patients vs. controls; lipid levels negatively correlated with age but not BMI or gender.
Stanciugelu et al., 2024 [60]	33 knee OA; 20 controls	Lipid biomarkers in plasma and SF for early OA diagnosis	UHPLC-QTOF-ESI+MS	Identified potential biomarkers such as PA 16:0/16:0, glucosylceramide, and PC 32:1 in plasma; PS and PI species more prominent in SF; disrupted pathways include plasmalogen synthesis and SL metabolism.
Li et al., 2024 [57]	45 OA; 111 RA; 25 controls	Lipid profiles in serum/urine for RA/OA diagnosis	UHPLC-ESI-MS/MS	Few lipids differentially expressed between OA and controls; SM t39:0 upregulated while LPC-O 18:0 down-regulated in serum of OA vs. controls; urinary lipid profiles less distinct for differentiating between groups.

This table condenses the studies’ focus areas, methodologies, and key findings for clarity and ease of comparison across the research presented in the attached document (ESI-MS/MS: electrospray ionization–tandem mass spectrometry; OA: osteoarthritis; LC-HRMS: liquid chromatography–high-resolution mass spectrometry; LC-MS/MS: liquid chromatography–tandem mass spectrometry; mod.: moderate; MS: mass spectrometry; SF: synovial fluid; UHPLC-ESI-MS/MS: ultra-high-performance liquid chromatography–electrospray ionization–tandem mass spectrometry; UHPLC-QTOF-ESI+MS: ultra-high-pressure liquid chromatography–accurate mass quadrupole time-of-flight mass spectrometry with electrospray ionization; UPLC-MS: ultra-performance liquid chromatography–mass spectrometry; UPLC-TOF-MS: ultra-performance liquid chromatography–time-of-flight mass spectrometry).

## Abbreviations

ABCA1: ATP-binding cassette transporter A1, ADMATS: a disintegrin and metalloproteinase with thrombospondin motifs, Apo: apolipoprotein, ASIC3: Acid-sensing ion channel 3, BMI: body mass index, BMP: bone morphogenetic protein, C1P: ceramide-1-phosphate, CEs: cholesteryl esters, Cer: ceramide, CH25H: cholesterol 25-hydroxylase, CYP7B1: 25-hydroxycholesterol 7-alpha-hydroxylase, COX-2: cyclooxygenase-2, DPPC: dipalmitoyl-phosphatidylcholine, FLSs: fibroblast-like synoviocytes, HDL: high-density lipoprotein, IL: interleukin, iNOS: inducible nitric oxide synthase, LDL: low-density lipoprotein, ESI-MS/MS: electrospray ionization tandem mass spectrometry, HA: hyaluronic acid, K-L: Kellgren-Lawrence, JNK: c-Jun N-terminal kinase, LC-MS/MS: liquid chromatography–tandem mass spectrometry, LPA: lysophosphatidic acid, LPC: lysophosphatidylcholine, LPE: lysophosphatidylethanolamine, LPS: lysophosphatidylserine, LRP3: Low density lipoprotein receptor-related protein 3, LXRs: liver X receptors, MAPK: mitogen-activated protein kinase, MMP: matrix metalloproteinase, MRI: Magnetic resonance imaging, MS: mass spectrometry, NHANES: national health and nutrition examination survey, OA: osteoarthritis, RORα: Retinoic acid receptor-related orphan receptor α, PC: phosphatidylcholine, PC P: phosphatidylcholine-based plasmalogen, PE: phosphatidylethanolamine, PE P: phosphatidylethanolamine-based plasmalogen, PG: phosphatidylglycerol, PGE2: prostaglandin E2, PI: phosphatidylinositol, PLA2: phospholipase A2, PLs: phospholipids, POPC: palmitoyl-oleoyl-phosphatidylcholine, PS: phosphatidylserine, RA: rheumatoid arthritis, S1P: sphingosine-1-phosphate, SF: synovial fluid, SLs: sphingolipids, SM: sphingomyelin, SphK1: sphingosine kinase 1, TNF-α: tumor necrosis factor α, TKR: total knee replacement, UHPLC-QTOF-ESI+MS: ultra-high-pressure liquid chromatography accurate mass quadrupole time-of-flight mass spectrometry with electrospray ionization, UHPLC-ESI-MS/MS: Ultra-high-performance liquid chromatography–electrospray ionization–tandem mass spectrometry, UPLC-MS: ultra-performance liquid chromatography–mass spectrometry, UPLC-TOF-MS: ultra-performance liquid chromatography–time-of-flight mass spectrometry, VEGF: Vascular endothelial growth factor, VLDL: very low density lipoprotein.
